# Recognizing megatsunamis in Mediterranean deep sea sediments based on the massive deposits of the 365 CE Crete event

**DOI:** 10.1038/s41598-022-09058-3

**Published:** 2022-03-28

**Authors:** A. Polonia, C. H. Nelson, S. C. Vaiani, E. Colizza, G. Gasparotto, G. Giorgetti, C. Bonetti, L. Gasperini

**Affiliations:** 1 Institute of Marine Sciences, National Research Council (ISMAR-CNR), Bologna, Italy; 2 CSIC, Instituto Andaluz de Ciencias de la Tierra, Granada, Spain; 3 Department of Biological, Geological and Environmental Sciences, University of Bologna, Bologna, Italy; 4 Dipartimento di Matematica e Geoscienze, University of Trieste, Trieste, Italy; 5 Universidade Federal de Santa Catarina, Florianopolis, Brazil

**Keywords:** Natural hazards, Marine chemistry

## Abstract

The Mediterranean Sea hosts two subduction systems along the convergent Africa-Eurasia plate boundary that have produced strong ground shaking and generated tsunamis. Based on historical descriptions and sedimentary records, one of these events, in 365 CE, impacted a broad geographical area, including tsunami evidence for distances of 700–800 km from the source event, qualifying it as a ‘megatsunami’. Understanding how megatsunamis are produced, and where they are more likely, requires a better understanding of the different secondary processes linked to these events such as massive slope failures, multiple turbidity current generation, and basin seiching. Our sedimentary records from an extensive collection of cores located in distal and disconnected basins, identify turbidites which are analyzed using granulometry, elemental (XRF), micropaleontological, and geochemical data in order to reconstruct their coastal or marine source. The results show that the 365 CE basin floor sediments are a mixture of inner shelf and slope materials. The tsunami wave produced multiple far-field slope failures that resulted in stacked basal turbidites. It also caused transport of continent-derived organic carbon and deposition over basal turbidites and into isolated basins of the deep ocean. The composition of sediment in isolated basins suggests their deposition by large-scale sheet like flows similar to what has been caused by the Tohoku earthquake associated tsunamis. This is significant for rectifying and resolving where risk is greatest and how cross-basin tsunamis are generated. Based on these results, estimates of the underlying deposits from the same locations were interpreted as possible older megatsunamis.

## Introduction

Recent large tsunamis in 2004 CE (Sumatra), 2010 CE (Maule, Chile) and 2011 CE (Tohoku-Oki, Japan) all took place along subduction systems and have shown that such events can be destructive across entire ocean basins. The Mediterranean Sea hosts two subduction systems (Hellenic and Calabrian Arcs) and has been the site of destructive earthquakes. The Calabrian Arc was struck repeatedly by moderate Mw = 7 tsunamigenic earthquakes^1,2^ that triggered late Quaternary turbidite deposition in the deep basins^3–5^. The Hellenic Arc is the most seismically active region in the Mediterranean Sea and was site of a M_w_ > 8 earthquake in 365 CE which generated a trans-Mediterranean tsunami^6^. The earthquake and tsunami of 21 July 365 CE has stimulated discussions mainly because historical records are not always concordant in chronology and extent of reported damages. Two different scenarios are proposed, the first with earthquake effects as restricted to Crete^7^ and the second with an unusual event with magnitude > 8.5^8^ with a tsunami spread across the Mediterranean Sea^6,9,10^. Such contrasting reports stimulate interest and witness large uncertainties about the consequences of such event in the Mediterranean Sea region. In recent decades the offshore sedimentary records have shown to have great potential for preserved tsunami deposits^11^ and useful information can be gleaned regarding event magnitude and recurrence^12^. The study of tsunami deposits, both offshore and onland, has increased exponentially following the devastating 2004 CE Indian Ocean^13,14^ and 2011 CE Tohoku-Oki tsunamis^15^. In areas with discontinuous terrestrial records and limited tsunami preservation, submarine deposits may contribute to improve tsunami-hazard assessment in the future^16^.

We analyze the sedimentary record of the most recent megaturbidite in the Ionian Sea, known in the literature as Homogenite/Augias turbidite (HAT)^17–20^. It was related to the Crete CE 365 Crete earthquake based on detailed radiometric dating^3,21,22^. However, the origin of the HAT remains an enigma because of the incongruity between its massive size (up to 25 m thick; > 150,000 km^2^ area) and the expectations of sediment transport from shallower areas due to earthquake disturbance in the Ionian Sea. The Italian-sourced part of the HAT cannot have generated the megaturbidite deposits from earthquake shaking and multiple slope failures, because the southern Ionian Sea slope sediment source is too far from the shaking energy of the earthquake rupture zone in Crete. Also there is no other italian earthquake causing this giant deposit^3^. Consequently our research question is: can the HAT megaturbidite result from shelf tsunami backwash and/or tsunami and seiche internal wave destabilization of the continental slopes? We analyse sediment samples collected in key areas of the Ionian Sea in an attempt to answer whether the HAT origin and sedimentary processes are related to the 365 CE tsunami.

## The Homogenite-Augias turbidite (HAT)

The Ionian Sea is filled by alternating pelagic sediment (including sapropel and tephra) and resedimented deposits. Megaturbidites are prevalent in the flat deep basin floor^3,4,22–27^ and they have been termed differently since their first recognition: turbidites^17^, homogenite^18^, unifites^28,29^, megabeds or mega-turbidites^30^. The uppermost of these beds in the near surface acoustic stratigraphic sequence, was named Homogenite/Augias Turbidite (HAT)^3^.

**Figure 1:**
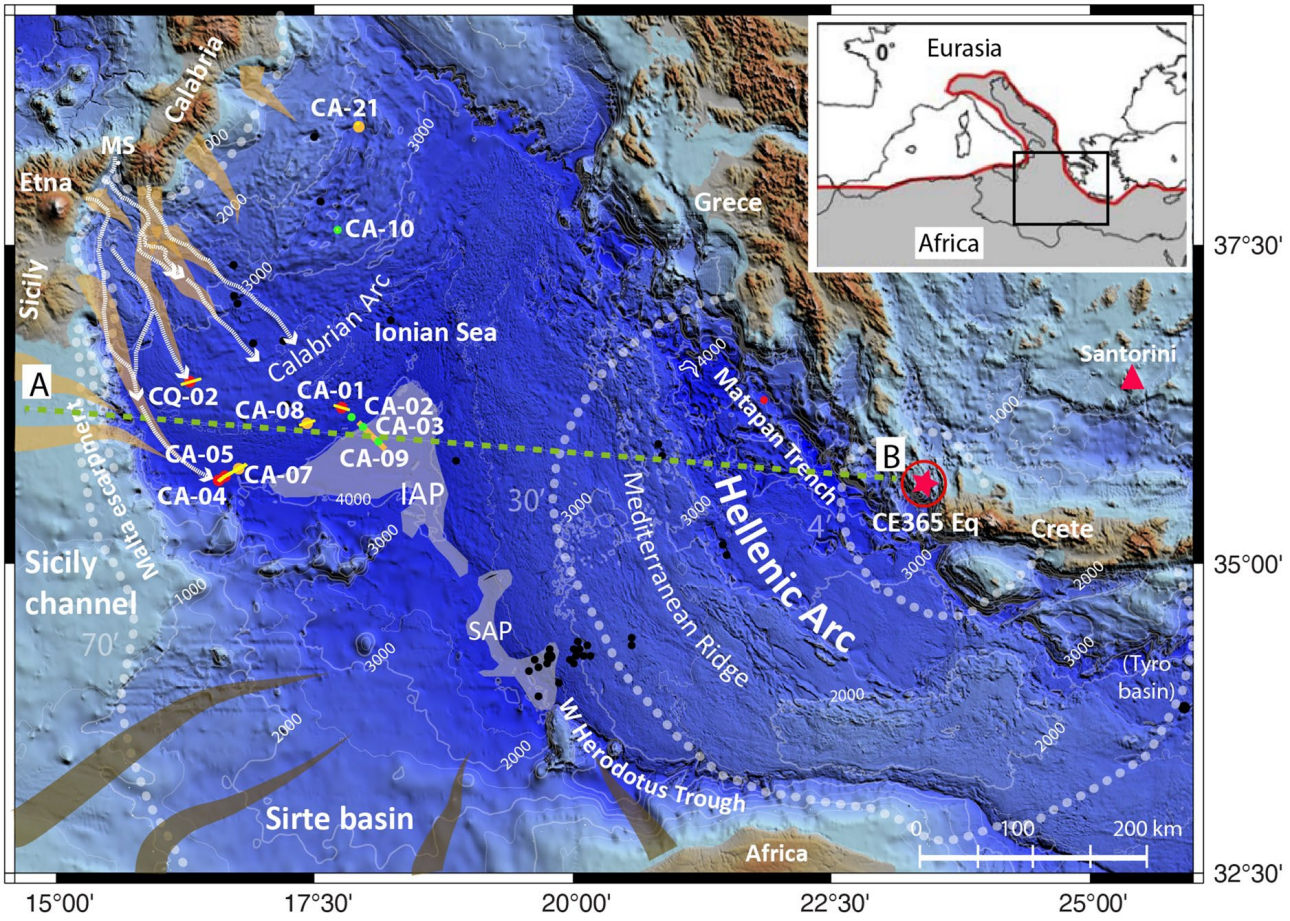
a: bathymetric map of the Ionian Sea with location of sediment cores CALA-01, CALA-04, CALA-05 and CQ14_02 (red dots) described in this study. Core CALA-05 was analysed in a previous study to define composition and structure of the HAT deposit^3^. Green dots represent cores described only in Fig. 7 in the correlation log. Yellow dots represent cores described in the Supplementary material (cores CALA-07, CALA-08, CALA-21: SM2-SM4). Black dots represent HAT occurrence in other cores^21^. Orange and brown lines: HAT sediment source areas^3,31^. A-B dotted green line: cross section in the conceptual model of Fig. 10. Yellow lines along the core transects: Chirp profiles in Fig. 2. Orange thick line across cores CA-03 and CA-09: Chirp profile in Fig. 11. White lines represent major canyon systems from the Messina Strait (MS) region down to the abyssal plain. Dotted grey lines show tsunami wave front at 4, 30, and 70 min after the earthquake^6^. Red star: epicentral area of the CE 365 earthquake. Name of cores is abbreviated. CA: CALA; CQ:CQ14. Inset map: the black rectangle represents the study region in the frame of the Africa (grey pattern)/Eurasia plate boundary (in red) in the Mediterranean region. Morphobathymetric data are from Global Bathymetry and Elevation Digital Elevation Model: SRTM30_PLUS v8 (https://data.gov.au/data/dataset/global-hillshading-from-srtm30_plus-v8-0-nerp-te-13–1-eatlas-source-ucsd/). The map was compiled using GMT package (version 6.0.0; ic-mappi ng-tools .org/), and the image was edited using Adobe Illustrator (CS6; https://www.adobe.com/).

The HAT was observed in a wide region of the Eastern Mediterranean (the western Mediterranean Ridge, Matapan trench and Calabrian Arc), in the Ionian and Sirte abyssal plains and in the Western Herodotus trough, where it attains a thickness exceeding 20 m^26,31^. For this reason, a sudden and catastrophic event that occurred in the Eastern Mediterranean, was invoked since the beginning of the studies of the HAT. A number of different catastrophic triggering mechanisms were originally proposed including the Santorini caldera collapse (3.5 ka)^18^. However, the HAT radiometric dating shows an age compatible with the CE 365 Crete earthquake^3,22^ (Fig. SM1) Further radiometric dating in an area greater than 150,000 km^2^, indicates that the different Mediterranean “Homogenite deposits” described in the literature are all synchronous and were deposited during a single basin-wide event within the time window CE 364–415 in good agreement with the Crete megathrust earthquake^21^. Correlation of the single-event HAT over a wide area, from the northern Ionian Sea, to the Mediterranean Ridge and anoxic Tyro basin South of Crete (Fig. 1), suggests that the CE 365 Crete earthquake and tsunami must have produced devastating effects in the Eastern Mediterranean Sea.

## Results and interpretations

**Figure 2:**
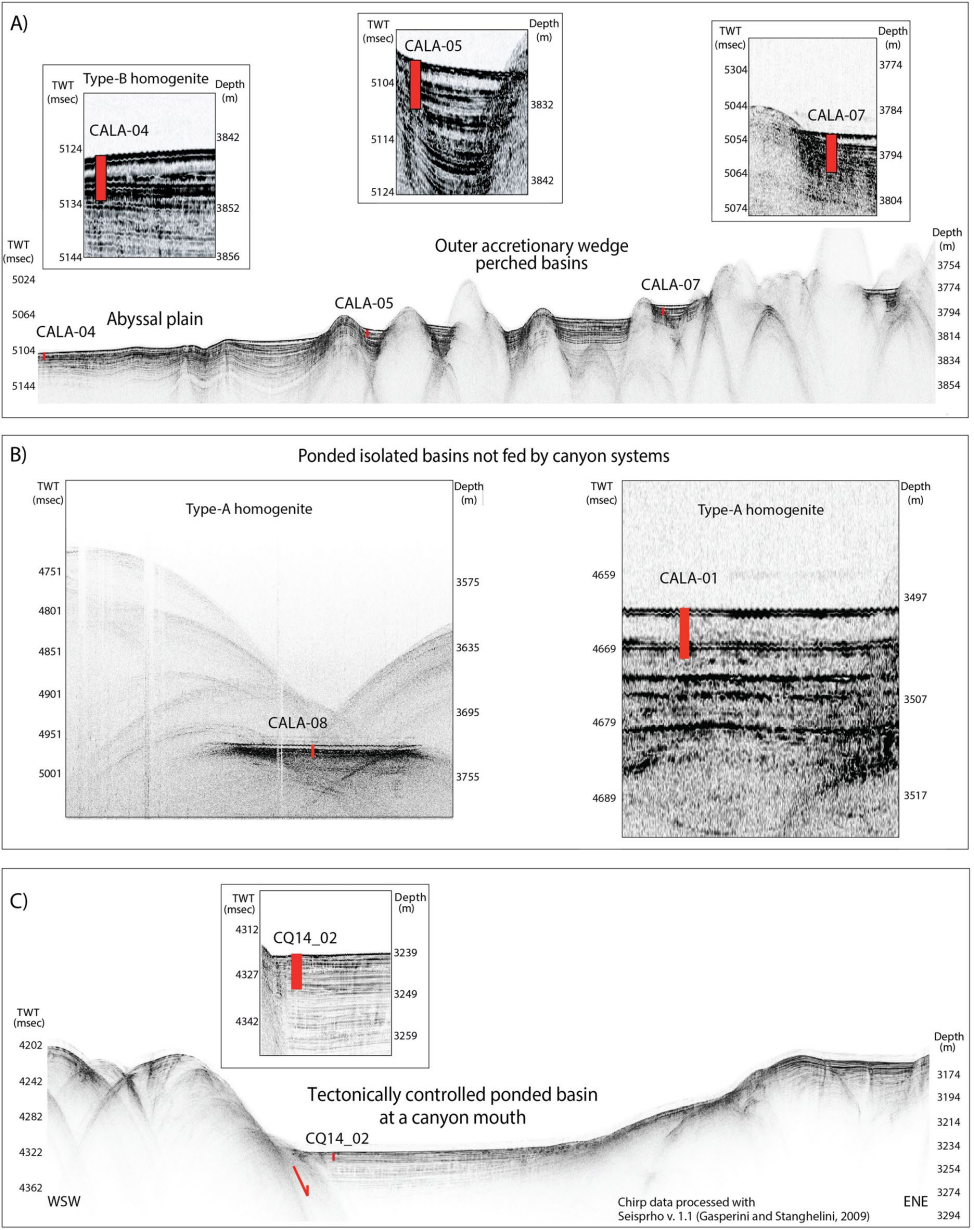
Sub-bottom CHIRP profiles across the coring sites investigated in this study (see Fig. 1 for location of Chirp profiles and gravity cores). (**A**) The Chirp profile across the core transect is collected at the transition between the undeformed abyssal plain and the accretionary wedge. Core CALA-04 represents the type-B homogenite^18^. (**B**) Chirp profile across the coring sites of CALA-01 and -08 in ponded isolated basins not fed by canyon systems (type-A homogenite^18^). (**C**) Chirp profile collected across the coring site CQ14_02 in the tectonically controlled slope basin directly fed by the canyon system from the Messina Straits region. Gravity cores are represented by red rectangles on the CHIRP profiles. Seismic data have been processed and geo-referenced using the open-source software Seisprho^72^.

**Figure 3:**
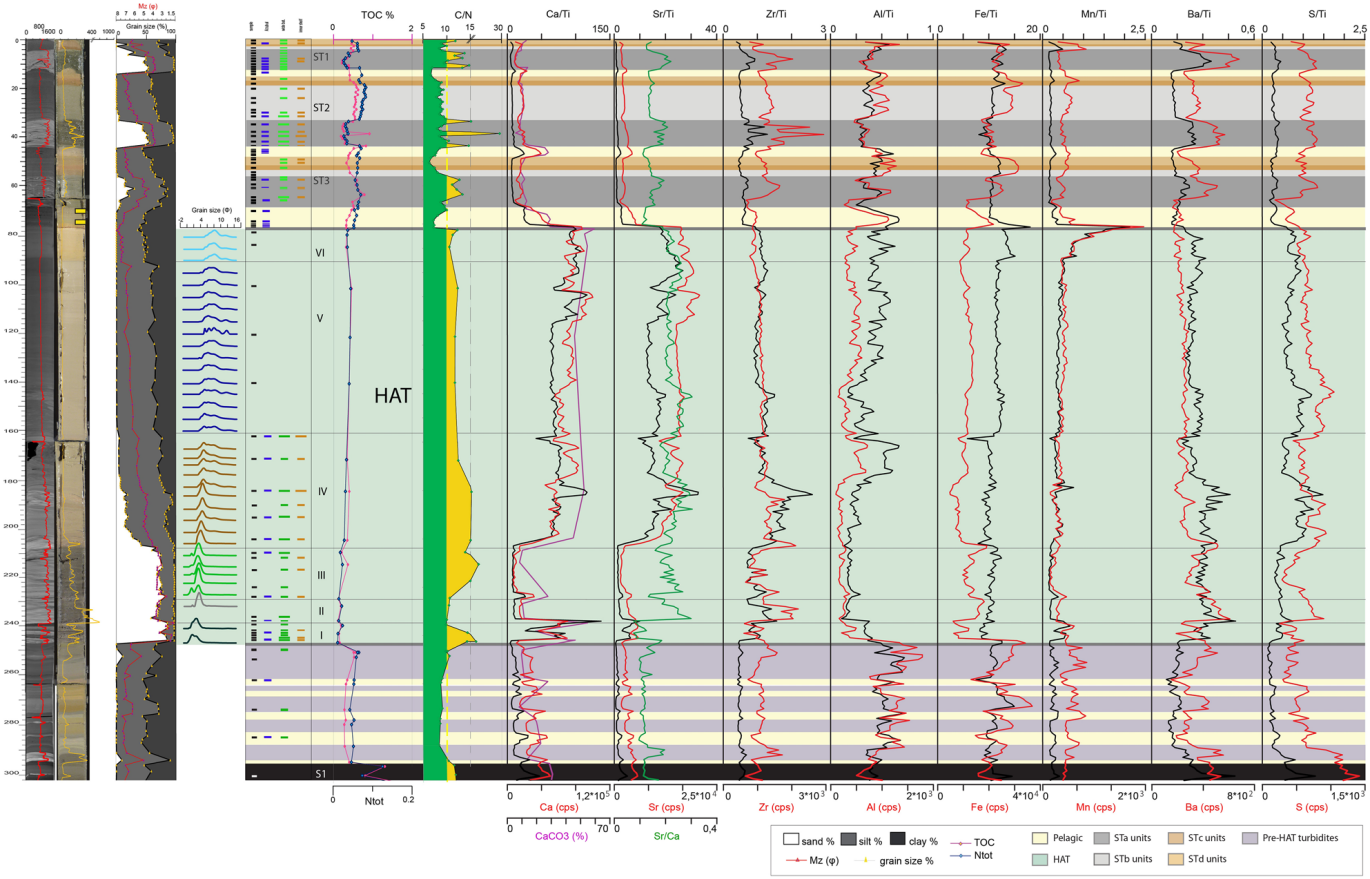
Log of core CALA-04. From left to right: CAT scan with HU (Hounsfield Unit) in red color, photograph with high-resolution magnetic susceptibility in yellow, grain size % with mean diameter in magenta, grain size modes in different colours for each sediment unit within the HAT, benthic foraminifera assemblages, sedimentary facies for ST1, ST2 and ST3 in different colors^4^, subdivision in individual HAT units (I-VI) in green and pelagic units identified by different colors, organic carbon data (TOC%, N_tot_, and C/N: green areas if C/N < 10, yellow areas if C/N > 10) and XRF data plot along the study sample. Yellow rectangle on the CAT scan image, represents ^14^C dated samples (SM1). Benthic foraminiferal assemblages are grouped in three different classes: blue = abyssal species (mainly A. tubulosa); green = wide bathymetric ranges; brown = inner shelf (see Table 2, SM6 and SM7 for more details). Thin sample rectangles represent 0,5 cm thick samples. Thick sample rectangles represent 1 cm thick samples. Small coloured rectangles = rare specimens: < 25 specimens in 0.03 g of sample; large coloured rectangles = common specimens: > 25 specimens in 0.03 g of sample (see Table 2 for details on the bathymetric distribution). Identified seismo-turbidites (ST1, ST2 and ST3) with individual units (a, b, c and d) are indicated.

**Figure 4:**
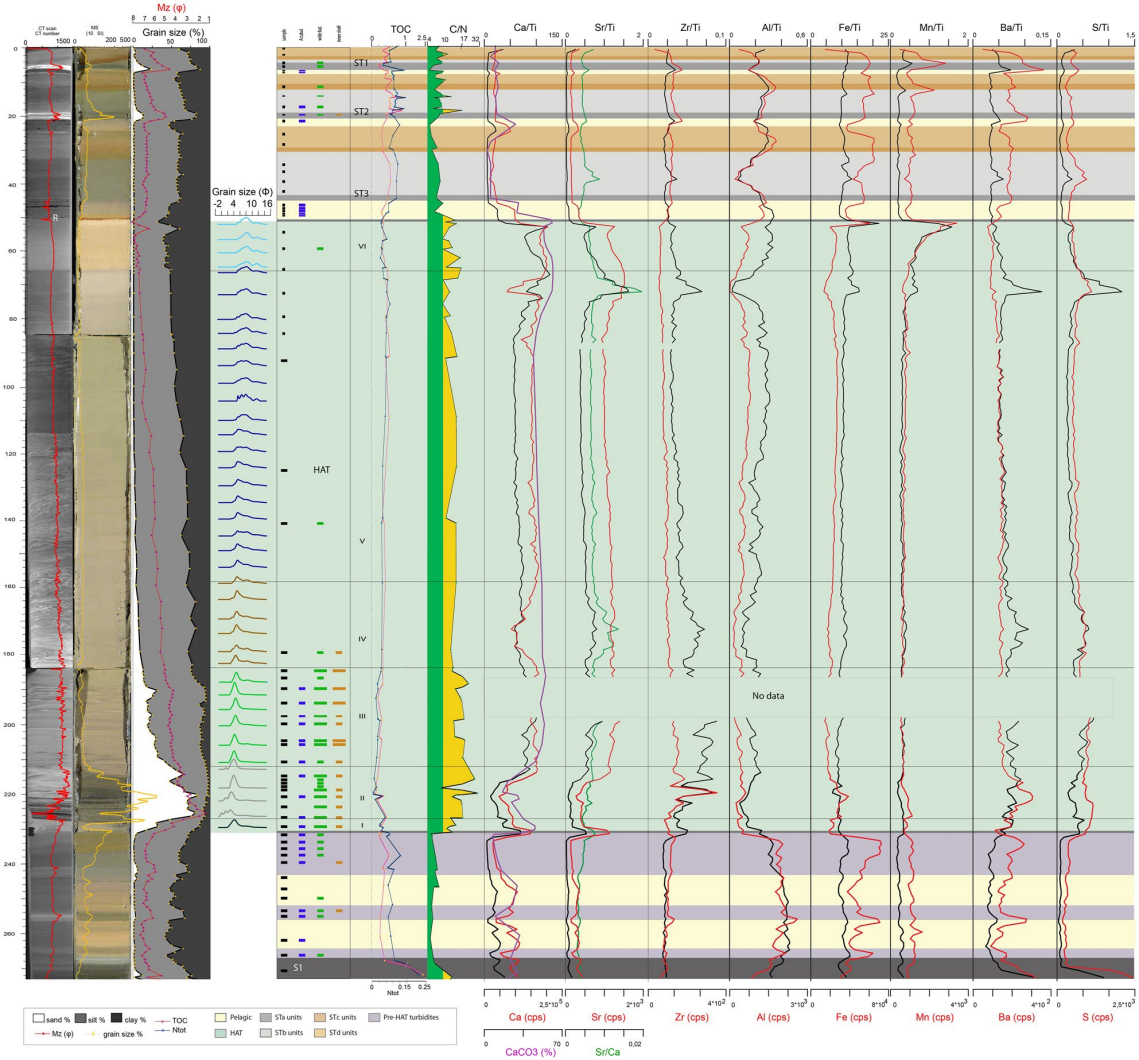
Log of core CALA-05. From left to right: CAT scan with HU (Hounsfield Unit), in red color, photograph with high-resolution magnetic susceptibility in yellow, grain size % with mean diameter in magenta, grain size modes in different colours for each sediment unit within the HAT, benthic foraminifera assemblages, sedimentary facies for ST1, ST2 and ST3 in different colors^4^, subdivision in individual HAT units (I-VI) in green and pelagic units identified by different colors, organic carbon data (TOC%, N_tot_, and C/N: green areas if C/N < 10, yellow areas if C/N > 10) and XRF data plot along the study sample. Yellow rectangle on the CAT scan image, represents ^14^C dated samples (SM1). Benthic foraminiferal assemblages are grouped in three different classes: blue = abyssal species (mainly A. tubulosa); green = wide bathymetric ranges; brown = inner shelf (see Table 2, SM6 and SM7 for more details). Thin sample rectangles represent 0,5 cm thick samples. Thick sample rectangles represent 1 cm thick samples. Small coloured rectangles = rare specimens: < 25 specimens in 0.03 g of sample; large coloured rectangles = common specimens: > 25 specimens in 0.03 g of sample (see Table 2 for details on the bathymetric distribution). Identified seismo-turbidites (ST1, ST2 and ST3) with individual units (a, b, c and d) are indicated. XRF data were published in a previous paper^3^.

**Figure 5:**
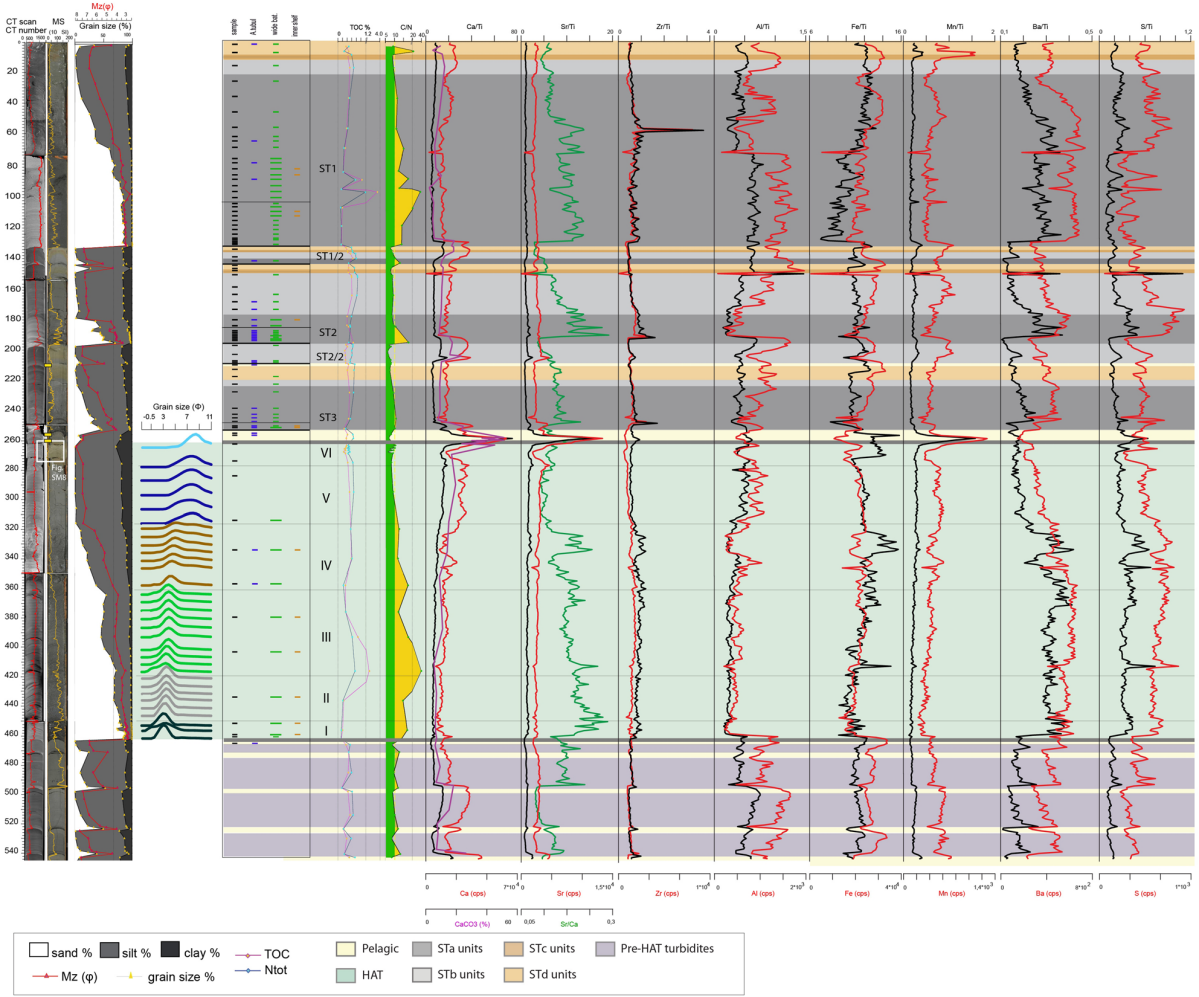
Log of core CQ14_02. From left to right: CAT scan with HU (Hounsfield Unit) in red color, photograph with high-resolution magnetic susceptibility in yellow, grain size % with mean diameter in magenta, grain size modes in different colours for each sediment unit within the HAT, benthic foraminifera assemblages, sedimentary facies for ST1, ST2 and ST3 in different colors^4^, subdivision in individual HAT units (I-VI) in green and pelagic units identified by different colors, organic carbon data (TOC%, N_tot_, and C/N: green areas if C/N < 10, yellow areas if C/N > 10) and XRF data plot along the study sample. Yellow rectangle on the CAT scan image, represents ^14^C dated samples (SM1). Benthic foraminiferal assemblages are grouped in three different classes: blue = abyssal species (mainly A. tubulosa); green = wide bathymetric ranges; brown = inner shelf (see Tables 2, SM6 and SM7 for more details). Thin sample rectangles represent 0,5 cm thick samples. Thick sample rectangles represent 1 cm thick samples. Small coloured rectangles = rare specimens: < 25 specimens in 0.03 g of sample; large coloured rectangles = common specimens: > 25 specimens in 0.03 g of sample (see Table 2 for details on the bathymetric distribution). Identified seismo-turbidites (ST1, ST2 and ST3) with individual units (a, b, c and d) are indicated. The white rectangle at about 260–270 cm depth refers to Fig. SM8.

**Figure 6:**
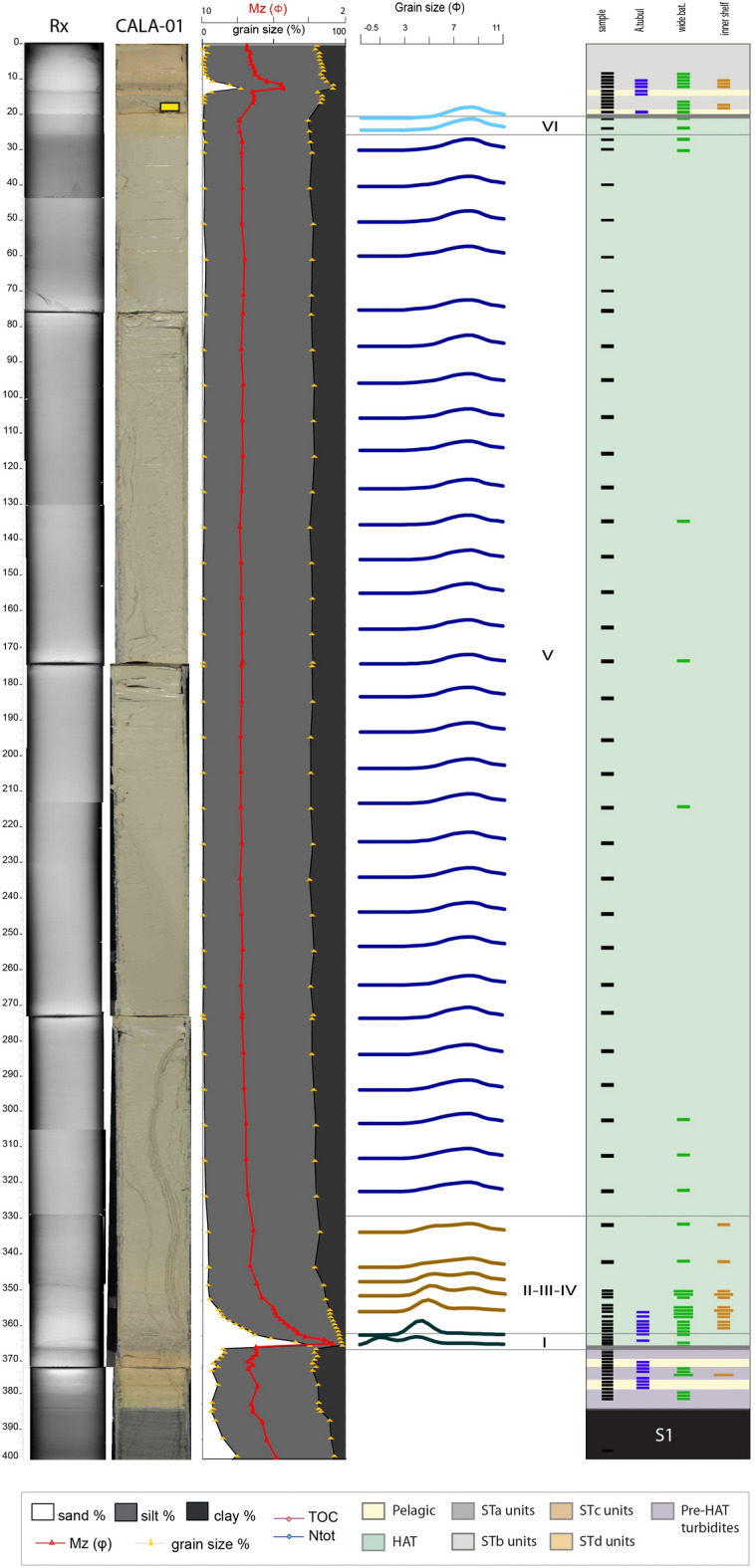
Log of core CALA-01. From left to right: CAT scan, photograph, grain size % with mean diameter in magenta, grain size modes in different colours for each sediment unit within the HAT, benthic foraminifera assemblages, subdivision in individual HAT units (I-VI) in green and pelagic units identified by different colors. Yellow rectangle on the CAT scan image, represents ^14^C dated samples (SM1). Benthic foraminiferal assemblages are grouped in three different classes: blue = abyssal species (mainly A. tubulosa); green = wide bathymetric ranges; brown = inner shelf (see Table 2, SM6 and SM7 for more details). Thin sample rectangles represent 0,5 cm thick samples. Thick sample rectangles represent 1 cm thick samples. Small coloured rectangles = rare specimens: < 25 specimens in 0.03 g of sample; large coloured rectangles = common specimens: > 25 specimens in 0.03 g of sample (see Table 2 for details on the bathymetric distribution).

We studied seven sediment cores collected in different basins (red and orange dots in Fig. 1 and marked in italics in Table 1) in order to provide information on sedimentary processes in different physiographic settings of the continental margin. The HAT deposit in core CALA-05 was described in a previous work^3^, while the other cores provide new data on sediment structure and composition. The settings range from the 4,000 m deep abyssal plain to shallower slope basins and structural highs up to 2400 m of water depth (Fig. 2). Core CALA-04 (Fig. 3) sampled the abyssal plain where it is fed directly by the westernmost canyon, which extends from the Mt. Etna region downslope along the base of the Malta escarpment (Figs. 1 and 2). Cores CALA-05 and -07 (Figs. 4 and SM2) sampled the perched basins of the outermost accretionary wedge that are close to the abyssal plain but not directly connected to the canyon systems. Core CQ14_02 (Fig. 5) sampled a ponded closed basin at a canyon mouth located in a slope terrace. Cores CALA-01 and -08 (Figs. 6 and SM3) sampled isolated slope basins between structural highs of the accretionary wedge including the Botticelli basin, where the HAT was first investigated^18,32^. These isolated basins have no connection with canyons. Core CALA-21 (SM4) was collected on a structural high offshore Calabria (Fig. 1) and is re-interpreted after previous work^24,33^.

**Table 1:** Total HAT thickness, thickness of sandy-silty basal Units –I to –IV, and thickness of uppermost Units-V and -VI (homogenite and laminite) for available gravity cores in the different depositional settings.

Oceanographyc setting	Core Id	Water depth	LAT	LON	Thickness (m)	Units I-II-III-IV (cm)	Units V-VI (cm)
						(% relative to the total thickness)	
**Abyssal plain margins and open basins**							
Confined Ionian abyssal plain close to Malta escarpment	***CALA-04***	***3907***	***35 39.643***	***16 34.845***	***1.82***	***87 (*~ *48%)***	***95***
Perched basin close to the abyssal plain	*CALA-05*	*3888*	*35 42.557*	*16 40.124*	*1.90*	*79 (*~ *41%)*	*111*
Perched basin close to the abyssal plain	***CALA-07***	***3880***	***35 45.159***	***16 45.834***	***1.80***	***65 (*~ *36%)***	***115***
**Abyssal plain (tabular hat)**							
Ionian abyssal plain	CALA-09	4043	35 57.991	18 06.990	> 5.20	Base not recovered	> 5.20
Ionian abyssal plain (previous studies*)					~ 12	NO DATA	> 120
Herodotus trough close to Cirenaica/Sirte(previous studies*)	LC-14	3574	33 23.840	20 30.230	~ 24	100 (~ 4%)	140
**Perched basins fed by canyon systems**							
**Canyons from the Strai of Messina**	***CORE CQ-02***	***3356***	***3556.6682***	***1604.44720***	***2.15***	***162 (*~ *75%)***	***53***
**Isolated disconnected depressions**							
	***CALA-08***	***3769***	***36 05.776***	***17 23.991***	***2.70***	***35 (*~ *13%)***	***235***
	***CALA-01***	***3812***	***36 14.044***	***17 46.270***	***3.47***	***36 (*~ *10%)***	***311***
	CALA-02	3808	36 09.848	17 51.760	1.55	15 (~ 10%)	140
	CALA-03	3968	36 05.120	17 57.849	2.50	15 (~ 6%)	235
	CALA-10	2991	37 36.927	17 43.511	1.65	23 (~ 14%)	142
	CALA-15	3070	37 50.050	17 33.713	2.55	41 (~ 16%)	214
	CALA-20	2171	35 41.566	16 37.954	2.60	70 (~ 27%)	190
	CQ14_01	3793	36 54.801	18 14.146	7.60	90 (12%)	6.7
**Structural highs**							
	*CALA-21*	*2396*	*38 24.994*	*17 55.671*	*0.05*	*1 (*~ *20%)*	*4*

Bold: cores discussed in this study that were not used to reconstruct HAT structure and composition before this study.

We used Chirp seismic profiles to estimate HAT thickness where sediment cores are not available and this was used to make a first order estimate for the total volume of the HAT resedimented material in the Mediterranean Sea. *: we integrated our analyses considering sedimentological information from the literature^19,22,33,64^. Cores marked in italics are shown in this manuscript.

### Sedimentary facies and sources

The identification of the HAT bed is based on: (a) sharp colour changes; (b) sharp basal boundaries, with coarse sandy basal units; (c) complex internal structure in the sandy/silty units with planar and cross bedding and possible convoluted layers; (d) multiple sand stacks with different composition; (e) characteristic biogenic content (such as *Posidonia oceanica*, inner shelf species of benthic foraminifera); (f) geochemical signatures such as C/N > 10 and high Sr/Ca associated with shallow water biogenic content (Figs. 3, 4, 5, 6).

**Figure 7:**
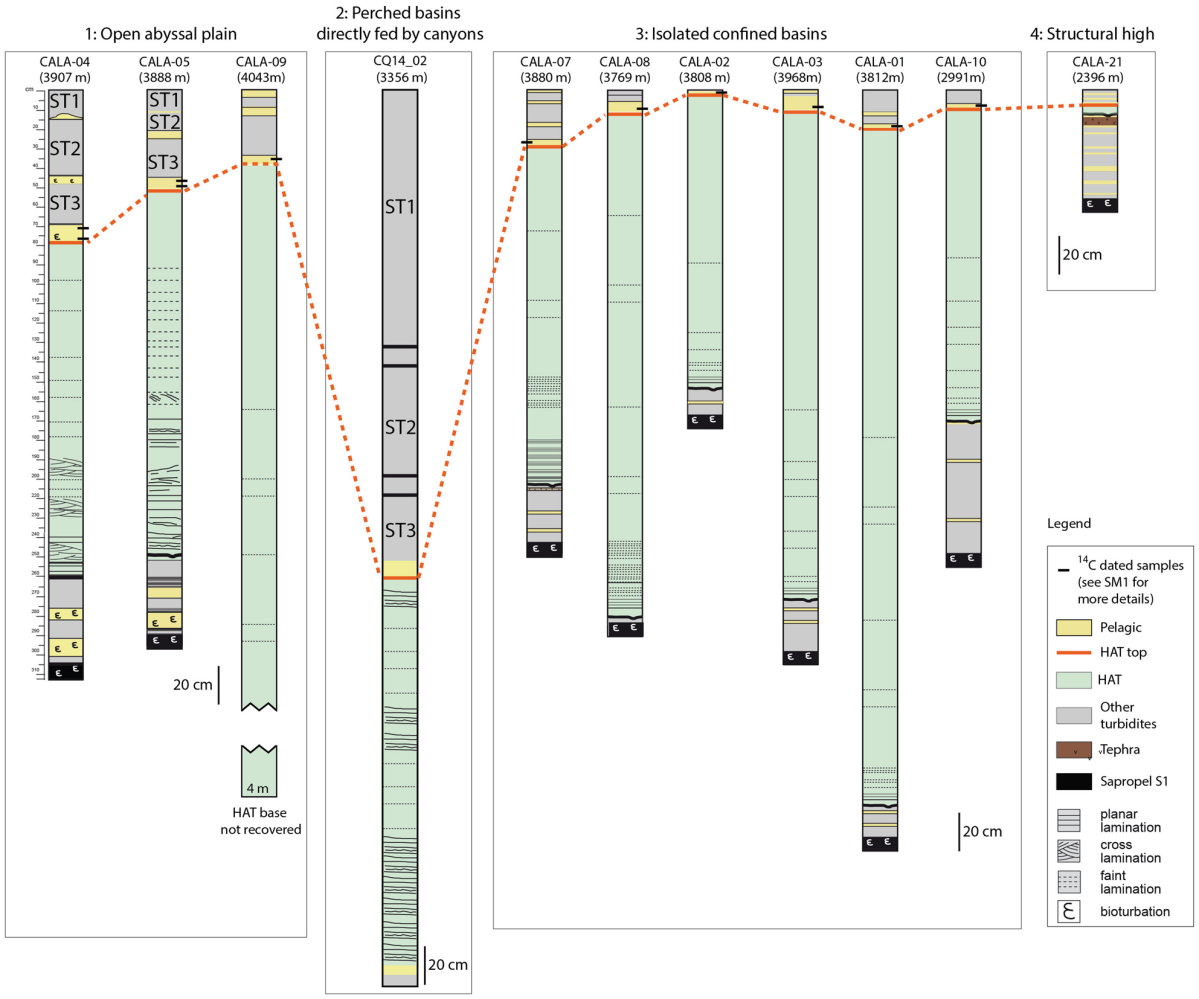
Correlation of the HAT thickness in gravity cores collected in different depositional settings. Open abyssal plain: CALA-04, CALA-05 and CALA-09. Perched basin directly fed by canyons from the Messina Straits region: CQ14_02. Isolated confined basins not connects to canyon systems: CALA-01, -02, -03, -07, -08, -10. Structural high: CALA-21 (Fig. 1 for core location). During the last 2000 yrs more than 90% of sedimentation in these cores is represented by seismically-triggered re-sedimented beds (green and grey units) while pelagic units (yellow) are thin layers bracketing turbidite beds. Pelagic sediment deposited after the 365 CE event were radiometrically dated (black rectangles) and radiometric dating results are discussed in Fig. SM1. The red line represents the top of the Homogenite/Augias Turbidite (HAT), which was emplaced after the CE 365 earthquake and tsunami^21^.

The HAT structure and composition suggest distinct sediment units (Table 1, SM5), but these have varying thickness, structure and composition in the different basin locations as evidenced considering stratigraphic correlation between 11 cores in the eastern Mediterranean (Figs. 1 and 7). Following, we summarise the sedimentary facies at each basin location.

#### Abyssal plain and basins connected with the abyssal plain (cores CALA-04 and -05, Figs. 3, 4, 7)

The different composition of the HAT units indicates different sources, sediment transport paths and sedimentary processes.

Units-I, -II and -III are sandy basal units where sand content is between 70 and 90% (Figs. 3 and 4). Unit-I is characterized by Ca-rich sediment with both deep water and inner shelf foraminifera (SM6-SM7). The source of Unit-I at these locations may be the Malta escarpment which is closest to the depositional site and where carbonate rocks outcrop along the continental margin^34^. Units-II and -III contain a composition high in Al, Fe, Ba, Zr and low in Ca suggesting a different sediment source. Both deep water and inner shelf foraminifera associations are present and terrestrial affinity is shown by C/N > 10 (Figs. 3 and 4). Considering the detrital components and minerals (quartz, feldspars and mica) a source region from the Calabrian low grade metamorphic basement or Mt. Etna volcanic region is proposed because these sediment source areas are connected to these sample locations via canyon systems (Fig. 1).

Units-IV and -V are generally fining upward sandy and muddy silt with Ca-rich sediment. In Unit-IV and -V there are places with increased sand inputs that are shown by the mean diameter (Mz) peaks and sedimentary structures (Figs. 3 and 4). Unit-IV is characterized by foraminiferal assemblages indicative of wide range of bathymetry. The sediment composition is a combination of both Malta Escarpment and Sicily sources and suggests that the source of this units is from the waning flows of multiple turbidity currents from different locations.

Units-V and -VI are characterized by foraminifera-barren silty mud (Figs. 3 and 4). Geochemical anomalies are present on top of Unit-V while C/N and grain size fluctuations (saw tooth profile of the silt/clay curves) characterize Unit-VI whose top is marked by prominent Fe and Mn peaks.

In summary, we interpret that Units-I to -IV are multi-source, stacked sandy turbidites related to synchronous slope failures and turbidity current generation. Unit-V is a thick homogenite bed, about 40% of the total HAT thickness, and sourced by slow settling down from the water column and waning of multiple turbidity currents (Figs. 3 and 4). The topmost Unit-VI appears to be sourced from sediment reworking by seiche water movements and chemical redox processes.

#### Ponded closed basin at a canyon mouth (core CQ14_02, Fig. 5)

The more uniform composition highlighted by the absence of strong elemental chemical variations in the different units suggests similar sediment sources or different sources but mixed together (Fig. 5). In this site that is sourced by a single canyon all units are low in Ca and show different composition relative to the other cores that are more affected by regional multi-sourced inputs.

Units-I, -II and -III represent the sandy basal units where sand content is fluctuating between 50 and 90% (Fig. 5). These units consist of Zr-rich sediments with high values of S, Ba and Cl, but low elemental concentrations of Ca and Al. This chemistry is accompanied by maximum C/N values at the base of Unit-III. Unit-IV is a fining upward silty mud unit but with sand input. At this depth, Cl and S anomalies and convoluted structures are accompanied by the presence of abundant *Posidonia oceanica*. Units I-IV include both deep water and inner shelf foraminifera (SM6-SM7). Units-V and -VI are homogeneous and barren clayey silt. Fluctuations of grain size and C/N values characterize Unit-VI whose top is marked by Fe and Mn peaks (Fig. 5, SM8).

In summary, basal Units-I to -IV are stacked sandy turbidite sourced by synchronous slope failures and turbidity current generation from low-Ca sources (Fig. 5). Unit-V represents the homogenite part of the turbidite bed which is related to the settling down from the water column and waning of turbidity current flows. At this location, the homogenite bed is about 20% of the total thickness. Ca elemental concentration slightly increases in this unit mainly from a biogenic component. In Unit-VI there is a very well preserved record of seiching structures as shown by sediment color changes and composition (SM8).

#### Isolated slope basins with no canyon connection (core CALA-01, Figs. 6, 7)

The HAT deposit in the isolated basin on the Calabrian Arc accretionary wedge, which is not connected to any canyon system, is different compared to the other locations and contains only four sediment units.

Unit-I in core CALA-01 (Fig. 6) exhibits a sand peak of about 70%; this sand, dominated by planktonic foraminifera without inner shelf benthic species, suggests a local, deep marine provenance (Fig. 6). Unit-II, -III and –IV are condensed and show an abrupt decrease in sand content, a fining upward trend and benthic foraminifera assemblages with both deep marine and displaced inner shelf species (SM6-SM7). Unit-V is thick and characterized by a barren and homogeneous muddy silt. The turbidite top of Unit-VI is characterized by color changes and the presence of a wide variety of bathyal benthic foraminifera species.

In summary, Unit-I represents reworking of sediment from a deep-sea source, while Units-II, -III and -IV has sediment from a continental shelf source. Units-II and -III sediment may be condensed in a single sediment unit or missing because there was not enough transport energy to deposit sediment in this isolated location without canyon systems. The homogenite Unit-V source is related to slow settling from the water column and waning of turbidity currents. The homogenite thickness is 85% of the total HAT deposit and may result from the confined basin setting that traps this sediment. The turbidite top is marked by an increase in Fe and Mn suggesting redox chemical conditions in this isolated basin setting.

#### Bathymetric high relief (core CALA-21, Fig. 7 and SM4)

Previous work has already reported the occurrence of the HAT on the higher elevation of structural highs in the Calabrian Arc subduction system^21,24^. Age models based on sedimentation rate^3^ and pelagic thickness between the tephra layer Z1 and the base of the HAT sediment have suggested that deposition took place in the time window 265–451 AD which agrees with the age of the HAT deposit in the rest of the Mediterranean Sea^21^. The HAT deposit on the structural high is less than 10 cm thick, marked by a sediment colour change and exhibits a sharp and irregular base associated with geochemical anomalies (high elemental concentrations of Zr and Mn and low Ca) (Figs. 7 and SM4). A mixture of sand grains and planktonic foraminifera characterizes the HAT sediment. Benthic foraminifera are rare and include species of a wide range of bathymetry, from outer shelf to abyssal environments (SM6-SM7). The occurrence of HAT deposits on structural highs 300–400 m above the surrounding plateaus suggests that widespread turbidity currents deposited the finer part of the HAT suspension flow throughout the entire Ionian Sea basin through un-channelized, sheet like sediment transport similar to the Japanese margin^35^.

### Statistical analyses of XRF data

Multivariate statistical analyses of the XRF-CS datasets for the CALA-04, CALA-05 and CQ14_02 provided further evidence on the relevance of the geochemical signatures for discrimination of sedimentary facies identified through these cores. Linear discriminant analysis (LDA) using ten XRF variables as predictors (Al, Ba, Ca, Cl, Fe, Mn, S, Sr, Zr and Sr/Ca) allowed to summarize the statistical differentiation between non-HAT deposits and the six HAT units (Fig. SM9). The overall accuracy of the LDA model was 72%. The non-HAT class, followed by the Unit-VI class, were the best discriminated ones, with the higher proportions of the samples correctly classified (92.6% and 86.7%, respectively). The first three basal units (Unit-I to -III) are relatively homogeneous among themselves (variation between the groups is small, while that within groups is large), but well differentiated from the topmost units (Unit-IV to -VI).

**Figure 8:**
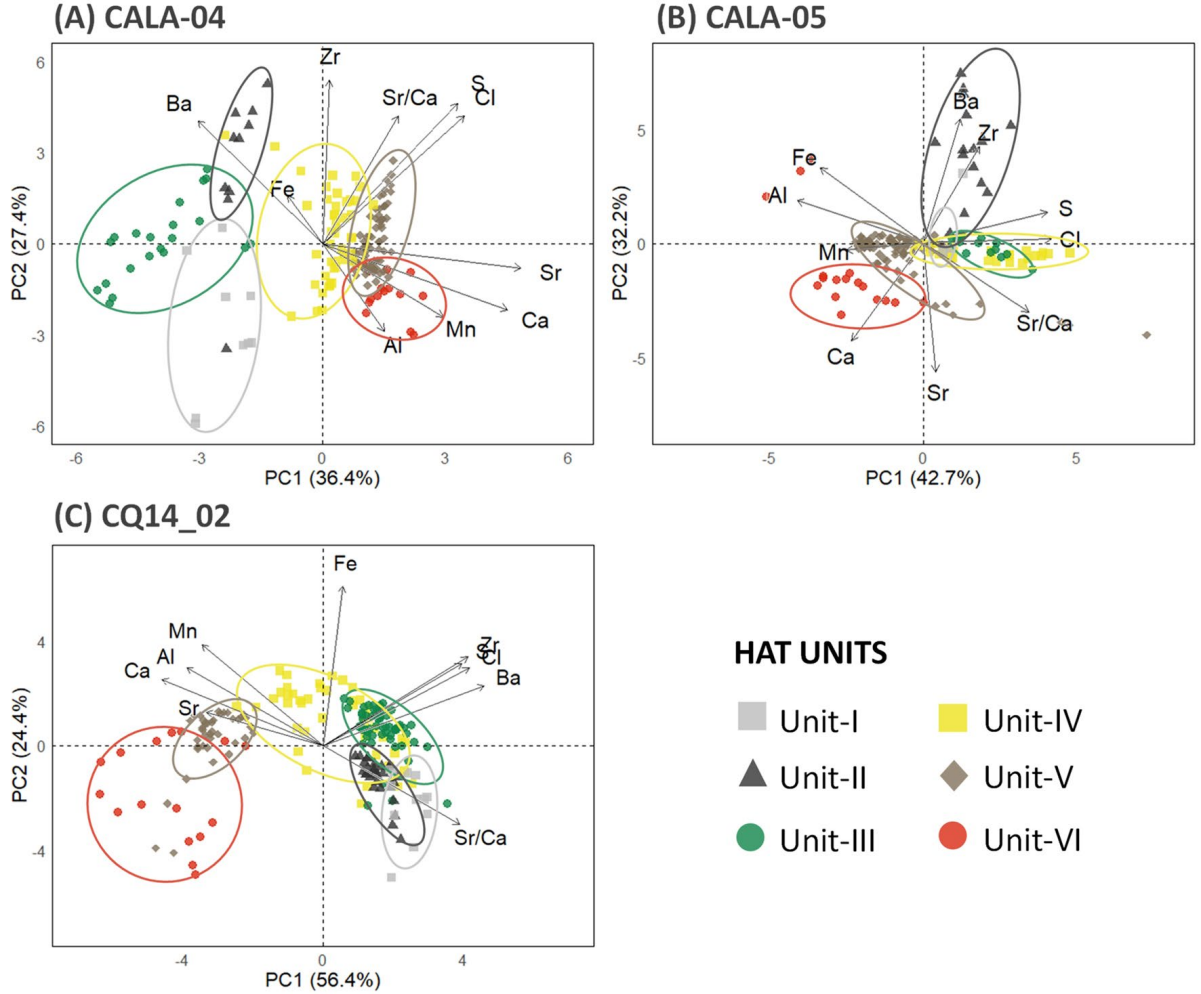
Statistical analysis based on XRF-CS datasets for the cores CALA-04, CALA-05 and CQ14_02. PCA biplots showing the contributions of 10 elemental concentrations to explain the HAT units in the core CALA-04 (**A**), CALA-05 (**B**) and CQ14_02 (**C**). The overlaps of the areas delimitated by the ellipses indicate higher similarities among the units, while the proximity of the units to the geochemical vectors suggest stronger contribution of them to differentiate the respective groups of the cases (1 cm vertical resolution samples). The percentages presented in the axis titles correspond to variance explained by each principal component (PC).

The correlation between HAT samples and the elemental concentrations used to discriminate the units were represented by the PCA biplots (Fig. 8). The three cores showed increases in Ca, Al, Mn and Sr values towards the top of the HAT deposit (higher concentrations in the units V and VI), while Ba and Zr values decreased. Besides this general pattern, the PCA performed for each core also allowed identification of specific differences between the variables that explain each HAT unit. These statistical differences suggest local variation in the depositional processes, as already explored in the previous sections.

### HAT geochemistry and sediment composition

The complex structure of the HAT is characterized by a varying biogenic and detrital composition in the different units. The core collected in a protected basin not fed directly by a canyon system (CALA-01, Fig. 6), does not contain inner shelf foraminifera in Unit-I, which are better represented in Unit-II and –III by small specimens (Figs. 1 and 6). Shallow water material in Unit-I is present only in those basins connected with canyon systems (CALA-04, CALA-05, CQ14_02) while in more isolated basin locations (SM2, SM3), the most basal sediment unit lacks shallow water material (Figs. 1, Figs. 3, 4, 5, 6, SM2, SM3). In basin locations connected with canyon systems and containing coarser sediment, other biogenic components are present. Shallow water material such as *Posidonia oceanica* (living at a shallow water depth in the range of 0–40 m), fish remains, inner shelf foraminifera, plant remnants, echinoids, Holothuroidea (*living at* 0–100 m water depth) are present in Units-II and -III.

Elemental ratio of Sr/Ca and all detrital content transported to the deep basin, combined with mineralogical analyses suggest a more continental affinity throughout the HAT deposit, which correlates positively with C/N ratios > 10 (Figs. 3, 4, 5, 6). The composition of the different HAT units is controlled also by sorting and post-depositional diagenetic processes. The content of Al seems subject to grain size control because the increase of Al correlates with the increase of medium to fine silt and decrease of Mz, which specifically characterize Units-IV and -V (Figs. 3, 4, 5, 6). Mn and Fe peaks in the topmost HAT Unit-VI agree with the paleo-redox classical model for gravity layers where Mn usually precipitates subsequently to Fe^36^. Sr/Ca is a good tracer for shallow water sediment when combined with micropaleontological analysis, total organic carbon data and C/N ratio. Peaks of Sr/Ca at the base of the HAT (for Mz > medium silt), associated with C/N > 10 in the same levels (basal units) (Figs. 3, 4, 5, 6) support the hypothesis of shallow water/continent-derived sediment sources^37^.

**Figure 9:**
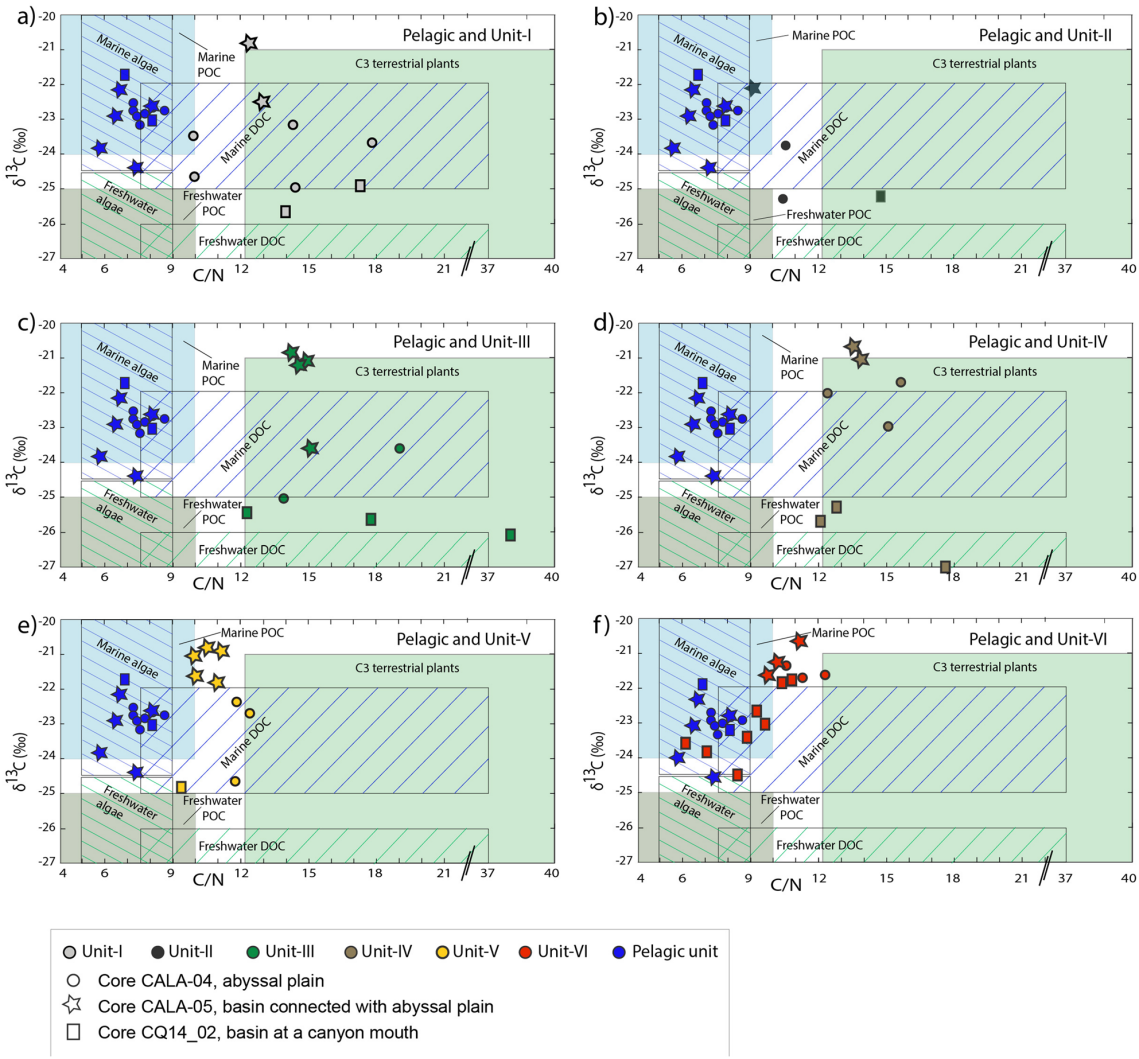
C/N versus δ^13^C_org_ for the HAT units in the three cores (circles: core CALA-04, stars: CALA-05, and rectangles: CQ14_02). (**a**) Pelagic and Unit-I samples; (**b**) Pelagic and Unit-II samples; (**c**) Pelagic and Unit-III samples; (**d**) Pelagic and Unit-IV samples; (**e**) Pelagic and Unit-V samples; (**f**) Pelagic and Unit-VI samples. The base diagram and environmental ranges are based on data established in previous studies to define the general d13C and C/N values that define coastal sediments^37^.

We have tested the terrestrial versus marine affinity through the analyses of C/N and δ ^13^C (Fig. 9). At all locations, C/N-δ^13^C plots show a marked difference between pelagic sediments (blue symbols) and HAT deposits, with the exception of Unit-VI in core CQ14_02. However, the three basin locations (CALA-04, CALA-05, CQ14_02) exhibit different isotopic and geochemical composition for the organic carbon. At the abyssal plain location of core CALA-04, the HAT shows more coastal affinity for Units-I, -II and –III, terrestrial plants affinity for Unit-IV and mixed or marine affinity for Units-V and -VI (Fig. 9). The HAT sediment in an open basin not directly fed by a canyon system (core CALA-05) contains a less abundant contribution from the coastal sources, while Units-III and –IV record a significant input from terrestrial organic carbon (Figs. 1, 4 and 8). In the ponded basin location of core CQ14_02, Units-I to -V include organic carbon of a coastal origin whereas the uppermost Unit-VI has more marine affinity (Figs. 1, 5 and 8). The terrestrial affinity is observed for samples in Units-III and –IV, which contain a great amount of *Posidonia oceanica* whose necromass is exported towards beaches and the terrestrial dune ecosystem^38^.

## Discussion

The expansion of the multi proxy dataset of stratigraphically preserved turbidites left by the 365 CE megatsunami has allowed a more detailed and comprehensive interpretation of the sedimentary processes that led to these deposits. Core analyses unraveled HAT’s geochemical, compositional and structure variations at abyssal depths and how these are related to secondary megatsunami processes.

### Sedimentary processes

The multi-sourced stacked turbidite beds of Units-I to -IV are interpreted to be near-synchronous (i.e. from the same event, but not necessarily at exactly the same moment) multiple sediment failures and turbidity currents that have travelled downslope from different sources and travel paths across the margin because they exhibit both terrestrial and marine sediment provenance (Figs. 3, 4, 5, 6, 8). The basal Units-I is present in all cores while Units-II to -IV are missing or condensed in isolated basins not fed by canyon systems (Figs. 6 and SM3). Unit V is the homogenite bed, which is present at all locations, and shows an expanded thickness in isolated basins (Table 1) where the suspension cloud of the turbidity currents is responsible for the majority of sediment deposition. The basin confinement traps the suspension cloud of the coeval turbidity currents and causes the slow deposition of the waning turbidite tail-homogenite muds over the basal coarser deposits. The homogenite thickness is minimum in cores directly fed by canyon systems (CQ14_02, Fig. 5 and Table 1) where traction processes prevail and deposition is not confined. Similar homogenite deposits have been described previously in many locations both in marine and lacustrine basins^39–44^. Homogenites are also found in the recent historic Ionian Sea seismo-turbidites deposited during major Calabrian Arc tsunamigenic earthquakes^4,5,23,45^, although none of these younger homogenites have the expanded thickness of the HAT deposit (Figs. 3, 4, 5,6).

At some locations above the homogenite deposit an alternation of white and brown layers with varying geochemistry are present (SM8). The white layers are high in Ca, Sr and Mn while the brown layers show an enrichment in detrital elements such as Ba, Al and Fe. These variations are accompanied by fluctuations in organic carbon with higher values of C/N in brown layers (SM8). Seiching bottom currents reworking sediment is the most likely process for this unit because there is no grading and multiple lamina are present suggesting forward and backward movement of material with different composition. The same type laminites on top of the homogenite deposit were previously identified for the CE 1908, 1693 and 1169 Calabrian Arc tsunamigenic earthquakes^4,5^. Laminites are associated with seismo-turbidites in the Marmara Sea^40^ and alpine lakes^39^ and also at the transition between the suveite and overlying Paleocene pelagic limestones in the Chicxulub impact crater where a megatsunami followed the impact^46^.

**Figure 10:**
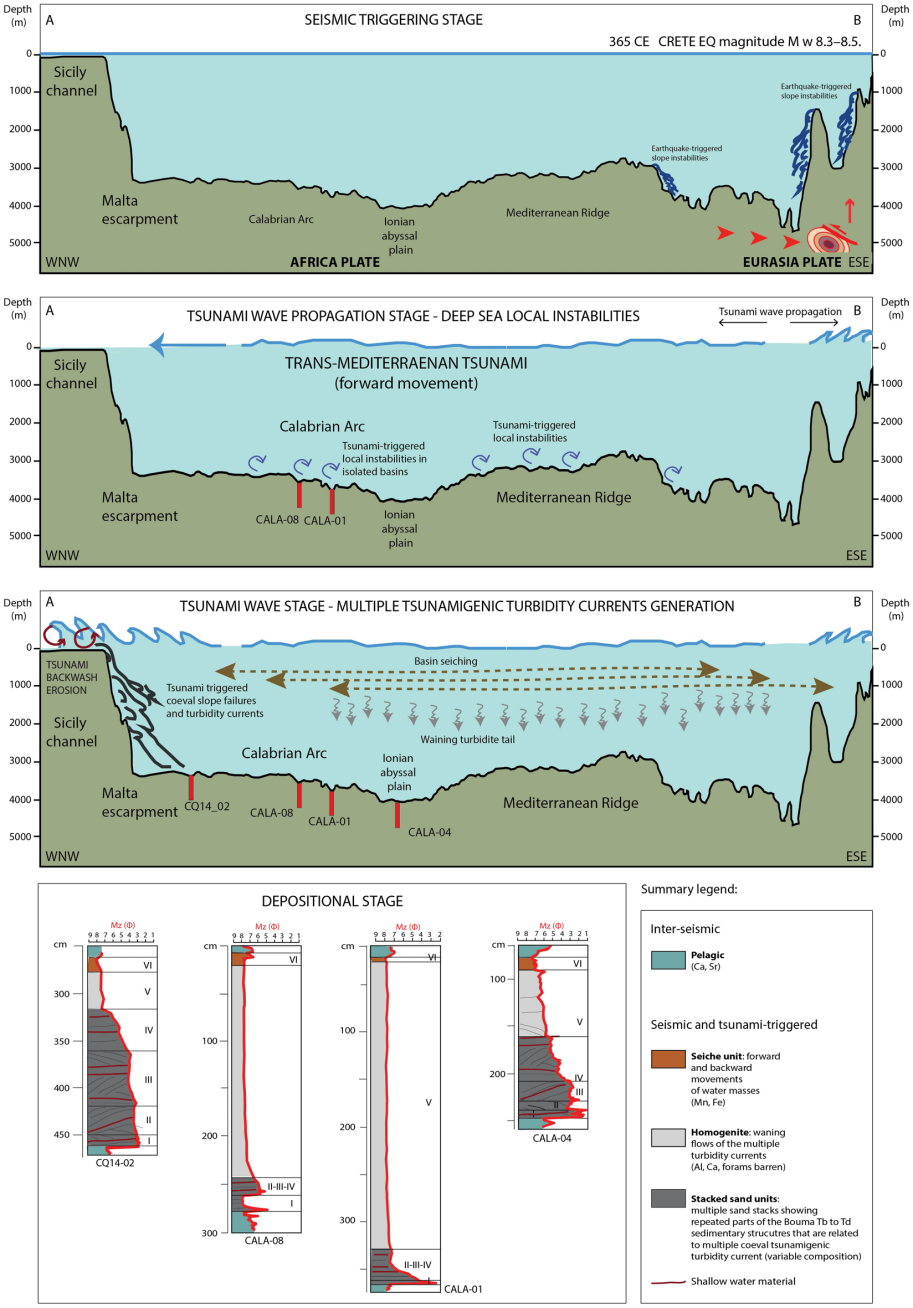
Bathymetric profile A-B across the Eastern Mediterranean Basin (see Fig. 1 for its location) and the conceptual model showing: (**a**) earthquake-generated processes; (**b**) propagation of a tsunami wave in the deep water generated by seafloor offset along a fault plane and by slope failures; (**c**) sedimentary processes related to multiple seismically triggered slope failures, resulting in multiple turbidity currents (black lines), waning turbidity currents (grey lines), seiching of confined basin water masses (brown dotted lines) and seafloor erosion of shoreline and shelf by tsunami wave backwash flows (red lines on the shelf); d) the resulting sedimentary processes deposits we observe in our cores in the different settings. Above the stacked sandy base (black), graded homogenite muds (grey), homogenite (grey) and laminites (brown). The figure was created using Adobe Illustrator (CS6; https://www.adobe.com/).

In summary we interpret the following processes and depositional patterns during the HAT deposition (Fig. 10). The lower depositional units of the HAT (Unit-I to –IV) are made up of stacked turbidites deposited by synchronously triggered multiple turbidity currents from different sources that transport shallow and deep-water sediment to depositional sites. Epiphytic foraminifers (such as *Asterigerinata mamilla, Rosalina* and *Lobatula lobatula*, see Table 2) are commonly observed in association with *Posidonia oceanica* and other inner shelf foraminifer species. This shallow water content for some of the Units I-IV sediment suggests a coastal and beach environment source and indicates that the tsunami caused substantial uprooting and seaward displacement of *Posidonia oceanica* with their benthic biota as observed offshore Eastern Sicily^11^. The occurrence of large fragments of *Posidonia oceanica* (SM10) in abyssal settings again suggest that HAT sediment was entrained from the coastal/beach environment where it usually accumulates during storm surges. A shallow water source is further supported by other sedimentological (grain size) and geochemical parameters (d13C, C/N, and detritic elemental concentrations).

**Table 2:** Water depth distribution of the main benthic foraminifera in core samples.

Inner shelf	Shelf and slope	Abyssal
Ammonia beccarii*	*Bolivina* spp.	*Articulina tubulosa*
*Asterigerinata mamilla*	*Bulimina* spp.	
*Biasterigerina planorbis*	*Cassidulina carinata*	
*Lobatula lobatula*	*Cibicidoides* spp.	
*Elphidium* spp.*	*Fissurina* spp.	
*Nonionoides turgidus*	*Gavelinopsis praegeri*	
Porosononion*	*Globobulimina affinis*	
*Quinqueloculina seminulum*	*Globocassidulina subglobosa*	
*Reussella spinulosa*	*Gyroidina* spp.	
*Rosalina* spp.	*Gyroidinoides* spp.	
*Spirillina vivipara*	*Lagena* spp.	
*Valvulineria bradyana*	*Melonis affinis*	
	*Pyrgo* spp.	
	*Quinqueloculina* spp.	
	*Trifarina angulosa*	
	*Uvigerina* spp.	

*Observed in cores Cala-04 and CQ14_02.

See the taxonomic reference list in Supplementary Material (SM6 and SM7).

Our results show that microfossils can be used to reconstruct the effects of tsunami waves. Previous studies in other areas also used foraminifera as a sediment source tracer and showed that large volumes of coastal material were transported seaward by tsunamis^11,47,48^. Nearshore benthic foraminifera (e.g., *Ammonia, Elphidium, Rosalina*) have been entrained by tsunami run-up and subsequently transported seaward by backwash where they were deposited in deep water sediments, which provides potential method to identify the sediment source of paleotsunami deposits^48^. However, displaced shallow water foraminifera are also characteristic of the basal part of classic turbidites that are not generated by tsunamis^49^.

Local sediment remobilization from basin walls to basin floors characterizes the basal part of the HAT deposits in small isolated basins (Figs. 6 and SM3). The composition of Unit-II in disconnected confined basins suggests that sediment entrained from inner shelf areas reached abyssal settings not fed by canyon systems (Figs. 1, 6, SM3). Sediment transport probably occurred through large-scale sheet-like flows similarly, to what has been proposed for the Tohoku earthquake^35^. Resedimentation processes were responsible for the deposition of the HAT not only in sedimentary basins but also in high standing plateaus and structural highs (CALA-21, Figs. 7 and SM4).

Deposition from a suspension cloud of multiple turbidity currents is responsible for the “homogenite” Unit-V. The HAT deposition of Unit-VI results from basin seiching which is responsible for the emplacement of the laminites Unit-VI (Figs. 3, 4, 5). The earthquake tsunami waves can cause oscillatory flow at deep-water depositional sites and also great earthquakes cause entire ocean basins to seiche. Seismic waves from the 1964 Alaska earthquake, for example, were so powerful that they caused water bodies to oscillate at many places throughout North America^50^. Thus, the depositional process may be one (tsunami oscillation) or the other (earthquake wave seiche) or a combination of both and we cannot clearly interpret the exact process. The millimetric black and reddish horizon enriched in Fe and Mn and with abundant Fe/Mn micro-nodules at the top of the HAT turbidite represents a diagenetic redox due to the mobilization of Fe and Mn within the turbidite which caused the development of a reducing condition induced by the rapid sediment accumulation of organic carbon-rich sediment and low sedimentation rate after the catastrophic event.

### Triggering mechanism

Basin-wide correlation of the single-event HAT suggests widespread massive sediment remobilization in the Eastern Mediterranean Sea (Fig. 1). It is not yet clear how the tsunami wave may have triggered such huge sediment remobilization and for this reason, we take into account both scenarios (seismic shaking and tsunami wave propagation) and discuss them in the light of the HAT composition and structure.

#### Seismic loading

Earthquakes may trigger sediment remobilization hundreds of kilometres from the epicenter but the sediment source and slope failures are generally close to the epicentral area depending on seismic loading. Shake maps of Mw > 8 earthquakes which have occurred worldwide since 2000 with a similar hypocentral depth to the Crete earthquake are generally associated with PGA (peak ground acceleration) between 0,1 and 1% g at about 500–600 km from the epicentre (Table SM11). These values are too low to trigger giant turbidity currents from continental margins so far from the epicentre^3^. It is generally recognized that 0.1–0.2 g PGA is the threshold of stability for the triggering of landslides in marine sediments^51,52^ and these values are much larger than those expected for a Crete-type earthquake in the HAT sediment source area at 500–600 km from the epicenter.

Sediment remobilization caused by liquefaction can also result from intense and long duration earthquake shaking^53^ and this depends on distance from the epicenter, soil type, initial confining pressure, and initial slope^54^. Liquefaction is the transformation of saturated granular material from a solid state to a liquid state as a consequence of increased pore pressures that reduces the effective strength of the material^55^ triggering surface disruptions and slope instabilities. The farthest liquefaction effects for a M_w_ = 8 earthquake is estimated to be in the order of 300 Km^56^. These data indicate that seismic shaking is unlikely to have triggered liquefaction in the study region.

#### Tsunami wave propagation

In confined basins not connected to any canyon system, the basal sandy Unit-I with a deep marine foraminifera assemblage suggests that local slumps were responsible for sediment remobilization into these isolated depressions (Figs. 6 and SM3). On the other hand, Units II-IV with inner shelf foraminifera in the same coring site (Fig. 6) indicates that shallow water material entrained from the coastal regions, took some time to be deposited, after the local slump. Based on these observations, the most likely scenario suggests that the basal local slump is related to stress changes during the forward propagation of the tsunami wave (Fig. 10). The shallow water Units II-IV material in isolated basins was remobilized when the tsunami front from the Crete earthquake impacted the Malta and Italian continental shelves and slopes and transported this material to the deep sea also in regions not fed by canyons systems (Fig. 10). The local slump may be related to liquefaction resulting from the pressure pulse exerted by the tsunami wave on the seafloor, similarly to that described for the nearshore environment^57^ or to tsunami triggered shear stress on the seafloor capable of suspending sediment as observed in shallow water^58^. The unusually steep continental slopes of the Malta and Italian margins may have aided the tsunami wave sediment remobilization processes (Fig. 1).

Previous sedimentological studies on the composition, lithology and volume of the HAT deposits^18,59^ suggested that it resulted from two simultaneous sedimentary processes: (i) Homogenite type-A local turbidites in small perched sedimentary basins with settling from a suspension of fine-grained sediment particles draping the basins walls; and (ii) distal turbidite type-B: deposition of a large and thick turbidite in the abyssal plains with provenance from the African continental margin. In this reconstruction, confined basins of the accretionary wedge were described as having only local provenance turbidites. Our study demonstrates that the HAT deposit contains material entrained from shallow water environments in every basin including the isolated basins and structural highs. This important observation suggests that the tsunami wave is capable of transporting large amount of shallow water material to the abyssal settings. Areas of locally thick HAT deposits (Figs. 6 and 7) have been sites of focused slumping, sliding and slope collapse possibly related to high relief steep slopes in the accretionary wedge and sediment remobilization related to tsunami waves. Thinning of the HAT deposits on structural highs indicates that these structures impeded thick sediment deposition there, but that depositional mechanisms were sufficiently energetic to overwhelm these features of regional positive relief.

Because Crete earthquake shaking could not have caused Italian margin failures, the most plausible explanation for large-scale sediment remobilization and terrestrial organic carbon transport to the deep basin during the CE 365 Crete event, are multiple large turbidity currents generated by slope failures when the trans-Mediterranean tsunami impacted the Italian and African continental shelves during its travel to the west from Crete (Figs. 1 and 9). Tsunami triggered turbidity currents also have been proposed for the 2011 Tohoku-Oki earthquake based on the displacement of an ocean-bottom pressure recorder after the main shock^60^. The presence of laminites at the top of the HAT deposit confirms water oscillation produced by a tsunami wave propagation as described in other regions worldwide^39,40^.

The tsunamigenic origin of the HAT is supported by coastal effects of the 365 CE Crete tsunami because resedimented deposits, erosional features and transported boulders with similar age, are reported from Egypt^61^ to Eastern Sicily^11,62^ and Malta^63^ around the Mediterranean Sea.

### Sediment volume

Before our study, the HAT unit was found only in basinal settings and not on the slopes or high standing plateaus, which separate the basins^18,32^. However, our study of a structural high close to the Calabrian coast, shows that the HAT deposit is present (< 10 cm thick) and has the same age (265–451 AD) as the HAT event (Fig. 7)^21^. Table 1 summarizes the total HAT thickness from available gravity cores. We used Table 1 data and Chirp seismic profiles (Fig. 2) to estimate HAT thickness where sediment cores are not available and this was used to make a first order estimate for the total volume of the HAT resedimented material in the study region of the eastern Mediterranean Sea (SM12). We integrated our analyses with previous sedimentological information from the literature^19,20,22,27,32,64^.

We divided the depositional area in the Calabrian Arc into seven polygons with similar homogenous seafloor physiography and HAT thickness (SM12). For each of these areas we have utilized a HAT characteristic thickness derived from the analyses of the reference cores (SM12). From these data, the total volume of the HAT in the Ionian Sea is estimated to be about 260 km^3^ with some uncertainties in polygon areas and HAT thickness estimates in the order of ± 10–20%. If we assume that sedimentary processes in the western Mediterranean Ridge are similar^59^ and considering that the Mediterranean Ridge area is twice that relative to the Calabrian Arc (Fig. SM12) this implies that the HAT volume in the Mediterranean Ridge is about 500 km^3^. Considering that the HAT volume in the Sirte abyssal plain, the Western Herodotus trough and the Matapan trench is estimated to be 24 ± 1 km^3^, 23 ± 1 km^3^, 5 ± 1 km^3^ respectively^27^, we consider a total HAT volume in the Calabrian Arc, abyssal plain and deep trench is a minimum of nearly 310 km^3^ with an additional HAT deposit on the Mediterranean Ridge for a possible maximum total of 810 km^3^.

### Recurrence time of catastrophic events in the Mediterranean region

**Figure 11:**
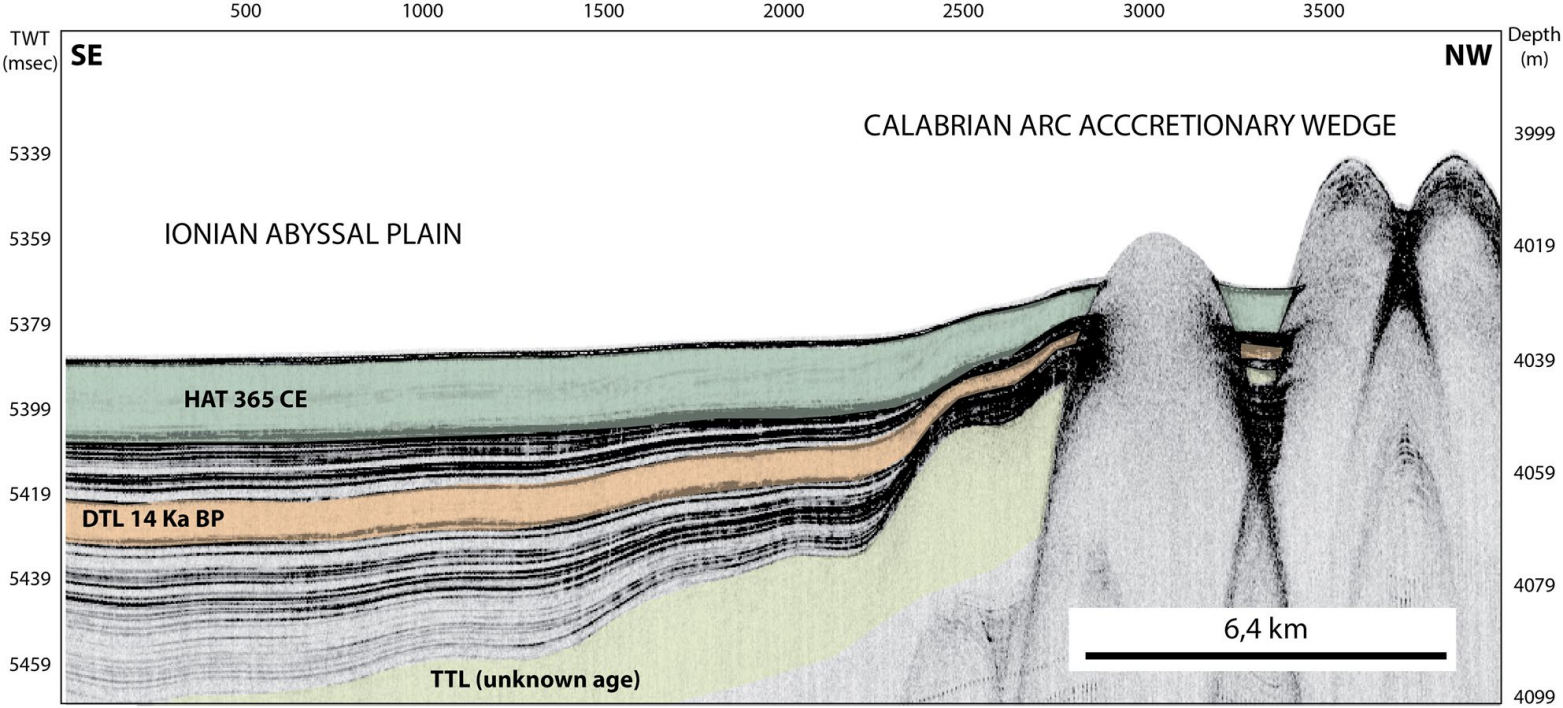
Chirp profile across the deformation front of the Calabrian Arc accretionary wedge (see location in Fig. 1). Three similar megaturbidites revealed by CHIRP data. The HAT (Homogenite-Augias Turbidite^3^), the DTL (Deep Transparent Layer^26^) and TTL (Thick Transparent Layer^26^) megaturbidites are highlighted in the profile by different colors (dark green, orange and light green, respectively). The low reflectivity seismic facies of these megabed in the Chirp profile is related to the upper rather homogeneous part of the megabed. The DTL composition suggests a similar source region as the HAT megaturbidite and an age of about 14 ka^3^. The age of the oldest megabed is still unknown. Seismic data have been processed and geo-referenced using the open-source software Seisprho^72^ and created using Adobe Illustrator (CS6; https://www.adobe.com/).

Paleo-shoreline observations in Crete suggest that the 365 CE earthquake is the largest event recognized in the historic past^6^. Also based on GPS deformation rates and estimates of co-seismic slip affecting marine terraces a ~ 4,500 year recurrence time for CE 365 type earthquakes has been suggested^6^. The Chirp profile in Fig. 11 suggests that two older turbidite megabeds are visible and can be correlated to the “Deep Transparent Layer-DTL” and “Thick Transparent Layer-TTL”^26^. AMS ^14^C ages from samples below the DTL megabed suggest it was emplaced after 14.590 ± 80 yrs BP uncalibrated age^3^. Considering the thickness of sediments between the DTL and TTL, we can speculate that the oldest megabed in our Chirp profiles is about 40–50 ka old. Between each megabed there are 7 and 21 minor turbidite deposits, which implies that the time interval between these megabeds is not constant.

We cannot exclude that the DTL and TTL were triggered by other mechanisms such as sea-level changes^65,66^ and gas hydrate phase change^67^ and more long cores are needed to go back in time and reconstruct the origin of such thick turbidites in the sub-seafloor. Defining sedimentological and compositional proxies for the last tsunamigenic megaturbidite provides the basis for the identification of previous trans-oceanic events in the Mediterranean region as has been done in other subduction zones worldwide^13,14,66–71^.

## Conclusions

New sediment cores help to define sedimentary processes during the propagation of the CE 365 Crete megatsunami and deposition of the HAT megabed on the deep-sea floor. The HAT deposit contains a volume of detrital siliciclastic and biogenic components as large as 800 km^3^, which were derived from the continental margins of the eastern Mediterranean Sea. The sediment from the European and African margins was remobilized and transported by tsunamis to abyssal depositional sites and isolated basins during the catastrophic event. The depositional units of the HAT deposit, when viewed across multiple geomorphological locations of the marine depositional sites (canyon mouth, abyssal, isolated higher basin), demonstrate a complex sequence of processes triggered by the megatsunami wave propagation.

The megabed consists of coarse basal stacked sand units, a homogenite, and an uppermost fine and laminated unit which result respectively from multiple slope failures, the waning flows of multiple turbidity currents and basin-wide seiche oscillations of the confined water masses. Sediment content from terrestrial and coastal sources, as shown by C/N and δ^13^C_org_ data and abundant shallow water components in isolated basins (*Posidonia oceanica,* benthic foraminifera such as *Ammonia, Elphidium, Rosalina*), indicates that tsunami backwash or tsunami triggered slope failure processes entrained coastal/inland sediment and transported it into the deep sea even in the absence of canyons, probably through large-scale sheet-like flows.

The HAT deposits could not have been triggered by the seismic shaking of the Crete earthquake, because shaking was not capable of triggering giant turbidity currents 500–600 km from the epicenter. A local turbidite bed at the base of the HAT in isolated basins on the accretionary wedge indicates that local sediment remobilization from basin walls was possibly related to the passage of the tsunami in deep water. When the tsunami wave hit the continental margins in Italy and Africa, it triggered turbidity currents on the slopes, which resulted in the stacked basal sand and silt units of the HAT. The analyses of geophysical data shows the presence of additional deposits of a similar nature from older layers with an age of about 14 and 40–50 Ka.

The HAT deposit demonstrates that a tsunami can trigger turbidity currents and the deposition of large sediment accumulations in deep-sea basins, which flushes significant volumes of sediment and organic carbon into the deep ocean, thereby affecting global geochemical cycling and deep-seafloor ecosystems.

## Methods

The core sections were analyzed through a multi-proxy approach involving geophysical, textural, geochemical, micropaleontological and compositional analyses (both biogenic and mineralogical). Results of radiometric dating obtained in previous studies were used to define a chronological framework.

Cores were collected during two cruises, CALAMARE08 (CALA-01, 04, 05, 07, 08, 21) and CALAQUAKE14 (CQ14_02), carried out onboard of R/V Urania in 2008 and 2014, respectively. During CALAMARE08 a 1.2 ton gravity corer was employed, with coring pipes 6 m long, while during CALAQUAKE14 cores were collected using a 2.3 ton CP20 piston corer, with coring pipes 10 m long. Cores CALA-07, -08 and -21 are shown in the Supplementary material (SM2, SM3 and SM4 respectively).

Chirp-sonar profiles were collected using a TELEDYNE-BENTHOS Chirp II system, with a 3–7 kHz operating frequency, and equipped with hull-mounted transducers (16 in total). Data were collected using a multi-ping technique, which allowed for an improved lateral resolution of the data. Chirp-sonar data were digitally sampled and stored in SEG-Y files. Quality-check and data processing, which included filtering and amplitude equalization, as well as statics and positioning correction, was performed onboard using the open ISMAR-CNR software SeisPrho^72^. Swath-bathymetry was collected using a SIMRAD 710 Multibeam echosounder.

Grain size was analyzed with a varying sampling rate depending on visual characteristics. Sediment texture analyses for cores CALA-04 and CALA-05 were performed through a coulter-counter laser Beckman LS-230, on the 0.04–2000 µm fraction. Core CALA-01, CALA-07, CALA-08 and CQ14_02, grain size analyses were performed by laser MALVERN Mastersizer 2000 (Hydro 2000S), for size ranges from 0.02 to 2000 µm fraction. Sediment samples were treated with 20 vol hydrogen peroxide solution for 48 h. Results were classified according to^73^ grain-size scale and are presented as % in sand, silt, clay and mean diameter (Mz) expressed in ɸ. Grain size and sedimentological data for core CALA-21 (SM4) was taken from previous studies^24,33^.

High-resolution Magnetic Susceptibility (MS) was acquired with a core logging system (Bartinghton model MS2, 100 mm ring) and acquisition was performed with a sampling interval of 0.5 cm.

X-Ray Computer Tomography (CT) was performed on all cores. Core sections were scanned by a medical CT system under X-Ray energy of 120 kV and pitch of 0.3. The final image have a voxel size of 118/512 (3.815*10–5) m^3^ and slice thickness of 1 mm. This method acquired projection images on different directions by application of a rotational movement of X-ray source and detector system on a steady sample. The intensity of the transmitted X-Ray beam is expressed as *Hounsfield Unit* (HU), which follow the relation: HU = (µ_m_- µ_w_) / (µ_w_) × 1000 where µ_w_ is the linear attenuation coefficient of the water, and HU depends on properties of the material (m) of X-ray absorption.

Analyses of planktonic and benthic foraminifera were performed on 214 samples from selected key layers. Samples, of about 3–8 gr of dried sediment, 0,5–1,0 cm thick, were dried at 44 °C for 24 h, weighed, soaked in water, wet sieved through sieves of 63 μm, dried and weighed again. Foraminiferal analyses were carried out on the size fraction > 63 μm. Concentration of benthic foraminifera was estimated in a split portion of 0,03 gr of dry residue > 63 μm, or on the entire sample if the size fraction > 63 μm is less than 0,03 gr. The identification of foraminifera and the ecological significance of benthic species was supported by selected key papers, as reported in^4^.

Mineralogical analyses were carried out on selected samples to define sediment composition and sources using Polarized Light Microscope (PLM), immersion liquids (RI = 1.60) and scanning electron microscope with EDS attachment. PLM allowed the identification of the main components (minerals and plant fragments), and SEM/EDS was used to identify minerals and estimate proportions among components.

Total carbon (TC) and total nitrogen (TN) were performed on selected homogenized sediments and determined using a *FISONS NA2000* elemental analyser coupled to a *Finnigan Delta Plus* mass spectrometer via a *CONFLO* interface. For measurement of the total organic content (TOC) and stable isotope ratios (δ^13^C_org_) of TOC, sediments were acidified (HCl, 1.5 M) to remove carbonates. TOC and TN contents are reported as percent of dry weight (wt%). C/N was calculated as molar ratio between TOC and TN (14/12*wt% TOC/wt% TN). The accuracy of element contents, calculated using an atropine standard, is ± 0.61 and ± 0.11% for carbon and nitrogen, respectively, while precision is 0.07 and 0.01% (1 std. dev.) Accuracy for δ^13^C and δ^15^N was ± 0.20 ‰ and ± 0.13 ‰ while precision was better than 0.2 ‰ (1 std. dev.). Determination of CaCO_3_ was done using this relationship: CaCO_3_ = 8.33*(TC%—TOC %)^74^.

Geochemical data of core CALA-05 were collected by using an *Avaatech* XRF-CS at GRC-University of Barcelona under two different settings, 10 kV (10 s measuring time) and 50 kV (30 s measuring time). CALA-04 and CQ14_02 were scanned using an *Avaatech* XRF-CS at ISMAR CNR-Bologna, under 10 kV (10 s measuring time), 30 kV (20 s measuring time) and 50 kV (30 s measuring time) settings. Measurements were performed with a step size of 1 cm along the cores. The XRF core scanner results are expressed as peak intensities in counts per second (cps) and also as normalization to Ti.

Multivariate statistical analyses were performed using 10 elements (Al, Ba, Ca, Cl, Fe, Mn, S, Sr, Zr and Sr/Ca) measured by the XRF-CS for the cores CALA-04, CALA-05 e CQ14_02. All analyses were conducted in the R software^75^. Linear discriminant analysis (LDA) was applied to assess the discriminatory ability of the elemental concentrations to predict seven depositional classes identified a priori as non-HAT, Unit-I, Unit-II, Unit-III, Unit-IV, Unit-V and Unit-VI. The LDA was carried out after the data partition in training and test datasets (0.7:0.3 ratio), and centering and scaling the elemental intensities. The confusion matrix represents how well the prediction data performs in assigning each sample to the given class. The data were processed using MASS R package^76^. Principal component analyses (PCAs) were run for the depth intervals correspondent to the HAT deposits in each core. The elemental concentrations were log-transformed to reduce the skewness. The factor scores of the two first principal components were plotted to explore the gradient of succession of the six lithological units identified through the HAT deposits, while loading vectors indicate the contribution of each individual variable toward the separation observed within the scores plot. The data analysis was performed in FactoMineR package^77^. All the plots were created in *ggplot2* package^78^.

## Supplementary Information

Supplementary Information.

## Data Availability

All digital data used for this work will be available at: ftp://ftp.ismar.cnr.it/outgoing/permanent_ro/HAT, an online data repository hosted at ISMAR-CNR.
