# Supplementary material for: Recognizing megatsunamis in Mediterranean deep sea sediments based on the massive deposits of the 365 CE Crete event
Source: Sci Rep. 2022 Mar 28;12:5253. doi: 10.1038/s41598-022-09058-3 (PMC8960809; doi:10.1038/s41598-022-09058-3)
Supplement: Supplementary file 1 — Supplementary Information. [file 41598_2022_9058_MOESM1_ESM.pdf]

Supporting Information for

Recognizing megatsunamis in Mediterranean deep sea sediments based on the massive deposits of the 365 CE event

Polonia A. (1)*, Nelson C. H. (2), Vaiani S.C. (3), Colizza E. (4), Gasparotto G. (3), Giorgetti G. (1), Bonetti C. (5), Gasperini L. (1)

[1] Institute of Marine Sciences, National Research Council (ISMAR-CNR), Bologna, Italy

[2] CSIC, Instituto Andaluz de Ciencias de la Tierra, Granada, Spain

3- Dipartimento di Scienze Biologiche, Geologiche e Ambientali, University of Bologna, Italy

4- Dip. di Matematica e Geoscienze, Universita' degli Studi di Trieste

5- Federal University of Santa Catarina, Florianopolis, Brazil

Contents of this file: Figures and Tables SM1 to SM12

SM 1 – Chronological framework: the Homogenite-Augias Turbidite (HAT) emplacement time

Accelerator mass spectrometry radiocarbon dating was performed on mixed planktonic foraminifera and pteropod fragments on 9 gravity cores in different regions of the eastern Mediterranean Sea (TableS SM1_1 and SM1_2). Radiometric dating results were published in Polonia et al. (2013a) and Polonia et al. (2016).

The first step to reconstruct the turbidite event chronology was to calibrate the ^{14}C ages of the pelagic sediment samples using the MARINE09.14 calibration curve (Reimer et al., 2009) in CALIB Rev. 6.0 programme (Stuiver et al., 2005) applying an average regional reservoir age of ΔR=0 and ΔR=147±33 calculated as the weighted mean including 2 ΔR values from published reservoir ages in the surrounding areas (Calib database at http://calib.qub.ac.uk/marine/) and considering that a mixing between Adriatic and Ionian deep water may be present in the working region. Results arising from the calibration are shown in Tables SM1_1 and SM1_2.

To determine the age of the HAT deposit we have used two different approaches:

A) Age model of two cores using radiometric dating of pelagic intervals above and below the resedimented deposit (Table SM1_1 and Fig. SM1A).

B) Radiometric dating of the pelagic interval deposited immediately after the catastrophic event in 9 cores (Table SM1_2 and Fig. SM1B).

We include here a summary of major findings deriving from these two approaches. Data and results described in this section are taken from previous studies (Polonia et al., 2013a; Polonia et al., 2016).

A) Oxcal age model of cores cala-04 and cala-05 as deduced in Polonia et al., 2013

C14 ages of pelagic sediment were used to perform an age modelling for cores CALA 04 and CALA-05 using the P_Sequence (a Bayesian model of deposition) implemented in the computer program OxCal 4.1; this software assimilates sedimentation as a random process following a Poisson law (Bronk Ramsey, 2008); marine data from [Reimer et al., 2009]. The modelling output is represented by the 95.4% probability age ranges (2σ) of each corrected depth corresponding to a turbidite. Turbidite beds, in fact, represent the "instantaneous sedimentary events" whose age can be derived through interpolation within the OxCal modelling.

We have built a depositional model with the OxCal software from the order of deposition of pelagic units and their depth derived subtracting the thickness of the turbidites from the total core. The software identifies mathematically a set of possible ages for each depth point in the sedimentary sequence (Supplementary 6 and 7 in Polonia et al. (2013)); turbidite age distributions at 2σ are modelled from their stratigraphic depth of emplacement into the background sequence (Fig. SM1A). The age intervals derived from the OxCal modelling are large because the P_Sequence is unable to narrow their time interval for lack of an adequate number of ^{14}C dated samples due to the scarcity of in-situ pelagic sediments suitable for dating. Despite this uncertainty, age modelling allowed us to deduce that the HAT age distribution is centred on the 365 CE Cretan earthquake (red line in Fig. SM1A).

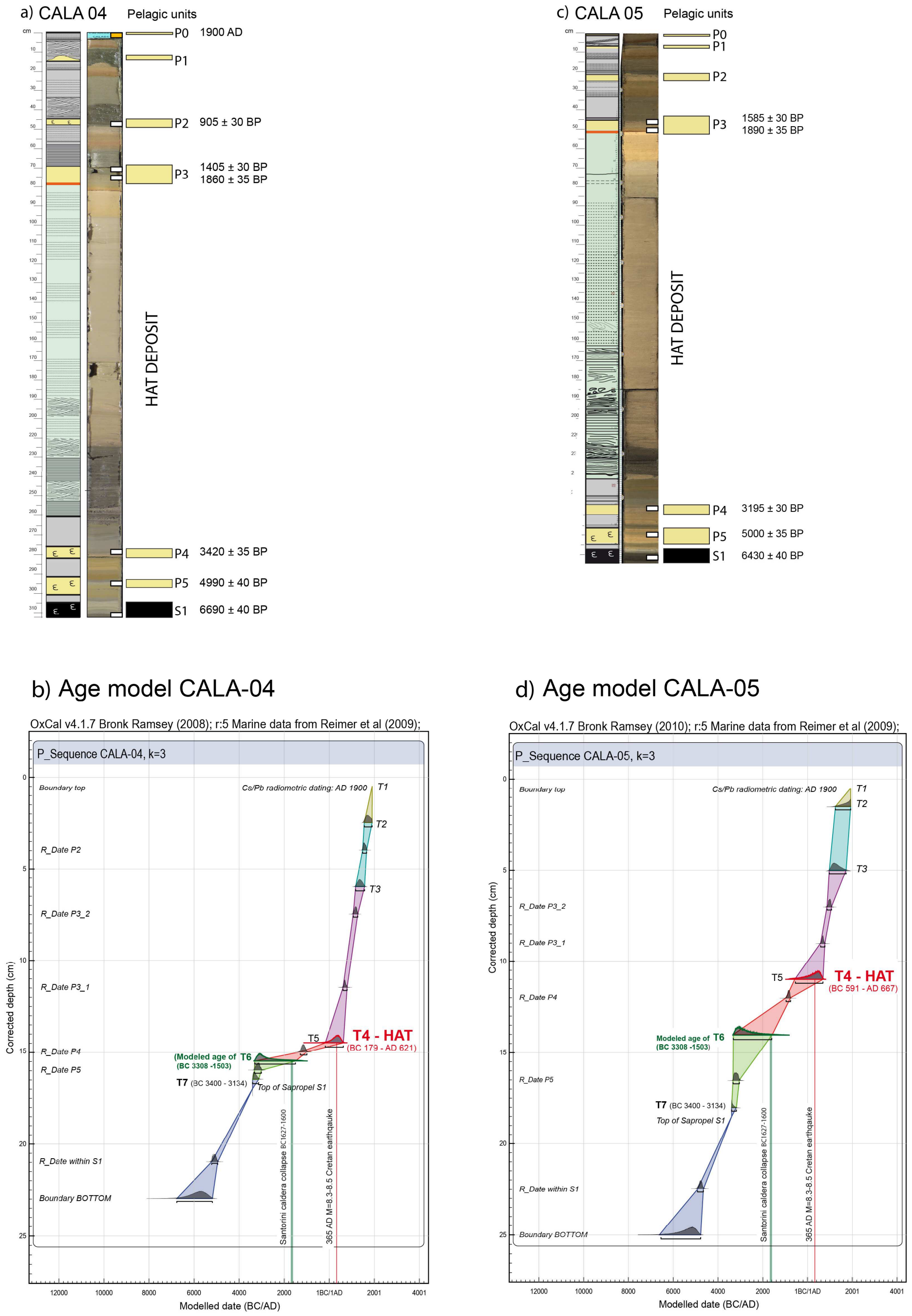

Fig. SM1A- *a) Stratigraphic log, photograph (14C and Cs/Pb dated samples are indicated by white and orange rectangles respectively) and pelagic units with uncalibrated radiometric ages of core CALA-04 (see Fig. 3 caption in the main text for symbols used in this stratigraphic log); d) age model built using the P_Sequence (a Bayesian model of deposition) implemented in the computer program OxCal 4.1 (Bronk Ramsey, 2008). See Polonia et al. (2013a) for more details. The regularity of sedimentation is determined by the k parameter (here k=3 reflects small variations in sedimentation rate as deduced from radiometric dating analysis). Modified from Polonia et al. (2013a).*

1<br>Core and water depth	2<br>Sample name and AMS Lab reference	3<br>Position relative to the HAT	4<br>Type of sampl e	5<br>14C age BP (uncalibrated)	6<br>Calibrated Age (2σ) according to CALIB REV5.0.2 by (*Stuiver et al., 2005)* ΔR=0	7<br>**HAT age with ΔR=0:** age interpolated on the top of the turbidite	8<br>Calibrated Age (2σ) with ΔR=147±33 (weighted mean including 2 ΔR in the region)	9<br>**HAT age with ΔR=147±33:** Age interpolated on the top of the HAT
**CALA-05 3814 m**	CALA-05 V 47-48 Poz-34625	4 cm above the top	Foram	1585 +/- 30	AD 717-894		AD 848-1055	
	CALA 05 V 49-50 Poz-38783	2 cm above the top	Foram	1890 +/- 35	AD 426- 610	**AD 81-399**	AD 560-762	**AD 215-547**
	CALA-05 III 42-43 Poz-34627	Within the coarse base	Foram	7340 ± 50	resedimented			
	CALA-05 III 45.5-47 Poz-34728	Within the coarse base	Foram	10770 ± 70	resedimented			
	CALA-05 III 61.5-62.5 Poz-35778	14 cm below the base	Foram	3195 +/- 30	BC 1170-1932		BC 982- 779	
	CALA-05 III 78-79 Poz-34933	32 cm below the base	Foram	5000 ± 35	BC 3496-3327		BC 3342- 3047	
	CALA-05 III 87-88 Poz-34934	41 cm below the base – within S1	Foram	6430 ± 40	BC 5091- 4834		BC 4932- 4667	
	CALA-05 I 37.5-38.5 Poz-34939	below the base of the DTL	Foram	14590 ± 80	BC 15647-14993		BC 15514- 14891	
**CALA-04 3845 m**	Cala 04 VI 45.5-46.5 Poz-35787	23 cm above the top	Foram	905 +/- 30	AD 1384- 1490		AD 1470- 1651	
	Cala 04 V 4-5 Poz-37402	7 cm above the top	Foram	1405 +/- 30	AD 970-1039		AD 1046- 1250	
	Cala 04 V 8-9 Poz-37403	3 cm above the top	Foram	1860 +/- 30	AD 452- 631	**AD 43-380**	AD 598- 781	**AD 189-530**
	CALA 04 IV 50 Poz-34629	Within the coarse base	Plant	4800 ± 35	resedimented			
	CALA 04 IV 99-100 Poz-37401	15 cm below the base	Foram	3420 +/- 35	BC 1397-1304		BC 1305- 1002	
	Cala 04 III 24-25 Poz-37398	39 cm below the base	Foram	4990 ± 40	BC 3499- 3311		BC 3337- 3023	

	Cala 04 III 38.5-39.5 Poz-37400	53 cm below the base (S1)	Foram	6690 ± 40	BC 5363- 5191		BC 5246- 4969	

Table SM1_1 *- Radiocarbon ages were obtained from planktonic foraminifera above and beneath the HAT, within the coarse base (green domain) and at different stratigraphic levels in two cores that contain the entire HAT sequence, CALA-04 and CALA-05 Modified from Polonia et al. (2013a).*

B) – Radiometric dating of the pelagic interval deposited immediately after the catastrophic event

The ages we obtained above the top of the turbidite and not just ontop of it, were corrected by the time interval corresponding to the thickness of pelagic sedimentation separating the top of the HAT and the dated level. For this purpose, we estimated the deposition rate from two successive radiometric dates in the pelagic sequence and we used this information to interpolate the age of the resedimented bed.

Pelagic sedimentation rates in the two cores have been estimated from two samples spaced 2 cm apart within pelagic unit P3 above the HAT in core CALA-05, and 4 cm apart in core CALA-04 (**Fig. SM1A**). The C14 ages of these samples suggest average rates of pelagic-normal sedimentation in the deep Ionian Sea of 0,068 mm/yr (0,054-0,083 mm/yr) in core CALA-05 and 0,097 mm/yr (0.095-0,100 mm/yr) in core CALA-04 (Polonia et al., 2013a). We derive that, under “normal” pelagic conditions, the time interval introduced by sampling above the top of the megaturbidite, is 110±46 and 140 ±46 years, for core CALA-04 and CALA-05 respectively, for each cm above the top.

1<br>Sample name and depth	2<br>Core depth (cm)	3<br>Position relative to the turbidite And type of sample	4<br>LAT	5<br>LON	6<br>Measured 14C age BP	7<br>Calibrated Age (2 σ) with ΔR=147±43 (weighted mean including 2 ΔR in the surrounding regions)	8<br>**HAT emplacement time with ΔR=147±43:** dates interpolated on the top of the turbidite
**CALA 04**	V<br>8-9	3 cm above the HAT top. Foram	35 39.643	16 34.845	**1860 +/- 30**	AD 578-786	**AD 189-530**
**CALA 05**	V 49-50	2 cm above the HAT top. Foram	35 42.557	16 40.124	1890 +/- 35	**AD 547-774**	**AD 215-547**
**CALA 01**	V 19-19,5	Just above the HAT top. Pteropods	36 14.044	17 46.270	2070 +/- 30	**AD 352-609**	**AD 352-609**
**CALA 02**	V 1-2	1 cm above the HAT top Foram	36 09.848	17 51.760	1629 +/- 29	**AD 789-1028**	**AD 633-964**
**CALA 03** (tot m)	VI<br>3-4	2 cm above the HAT top. Foram	36 05.120	17 57.849	1670 ± 28	**AD 744-998**	**AD 432-870**

**CALA 07** (tot m)	V 28-29	1 cm above the HAT top. Foram	35 45.159	16 45.834	1957 ± 27	**AD 464-686**	**AD 308-622**
**CALA 08** (tot m)	V 6-7	2 cm above the HAT top. Foram	36 05.776	17 23.991	1792 ± 28	**AD 655-874**	**AD 343-746**
**CALA 08** (tot m)	V 6-7	2 cm above the HAT top. Pteropod	36 05.776	17 23.991	1771 ± 28	**AD 669- 889**	**AD 357-761**
**CALA 09** (tot m)	VI 34.5-35.5	3 cm above the HAT top. Foram	35 57.991	18 06.990	1644 ± 27	**AD 780-1021**	**AD 222-739**
**CALA 09** (tot m)	VI 34.5-35.5	3 cm above the HAT top. Pteropod	35 57.991	18 06.990	1836 ± 29	**AD 602-816**	**AD 44-534**
**CALA 10** (tot m)	IV 9.5-10.5	2 cm above the HAT top. Foram	37 36.927	17 43.511	1670 ± 35	**AD 736-1002**	**364-814**

Table SM1_2 *- Accelerator mass spectrometry (AMS) radiocarbon dating of pelagic sediment above the HAT deposit. Accelerator mass spectrometry (AMS) radiocarbon dating was performed on mixed planktonic foraminifera (11 samples) and pteropod fragments (2 samples) collected ontop of the HAT deposit. Approximately 40-70 mg of pristine planktonic foraminifera or perfectly cleaned pteropod fragments, with no evidence of abrasion or carbonate overgrowth were handpicked in the size fraction > 250 µm in the first available pelagic sample above the HAT including sufficient material for radiocarbon dating. Modified from Polonia et al., 2016.* ^{14}C *ages (Poznań Radiocarbon Laboratory - Foundation of the Adam Mickiewicz University, Poland) for CALA samples analyzed in this study. Measured ages were calibrated according to the radiocarbon calibration program CALIB REV6.0.0 (Stuiver et al., 2005) and results are reported for ΔR=147±43 (column 7) calculated as the weighted mean including 2 ΔR values from published reservoir ages in the surrounding areas (Calib database at http://calib.qub.ac.uk/marine/). The age of the HAT (column 8) was obtained considering the time span corresponding to the thickness of pelagic deposits between the top of the HAT and the dated level. Modified from Polonia et al., 2016.*

Radiometric dating (Fig. SM1B) indicate that the narrowest emplacement time window for the turbidite is CE 364–415 with the exception of two samples from two different cores indicating a younger age (CALA-02 and CALA-03, Fig. SM1B).

Results from this study indicate that, within the errors associated with radiometric dating, HAT emplacement time is the same in the different basins of the Mediterranean Sea. This supports the idea of a single basin-wide event, able to re-suspend sediments over a very wide region including the Mediterranean Ridge, the Ionian abyssal plain, the Tyro Basin and the Sicily and Calabria slopes (Polonia et al., 2016). The HAT was radiometrically dated over an area of more than 25,000 Km^2, and considering chronologies recorded in the CR and MR integrated with revised age modeling in the Tyro Basin (Fig. SM1B) the area might increase to over 150,000 Km^2. The process that caused this huge event, was thus capable of synchronously triggering sediment remobilization in Crete, northern Africa, Calabria and Sicily.

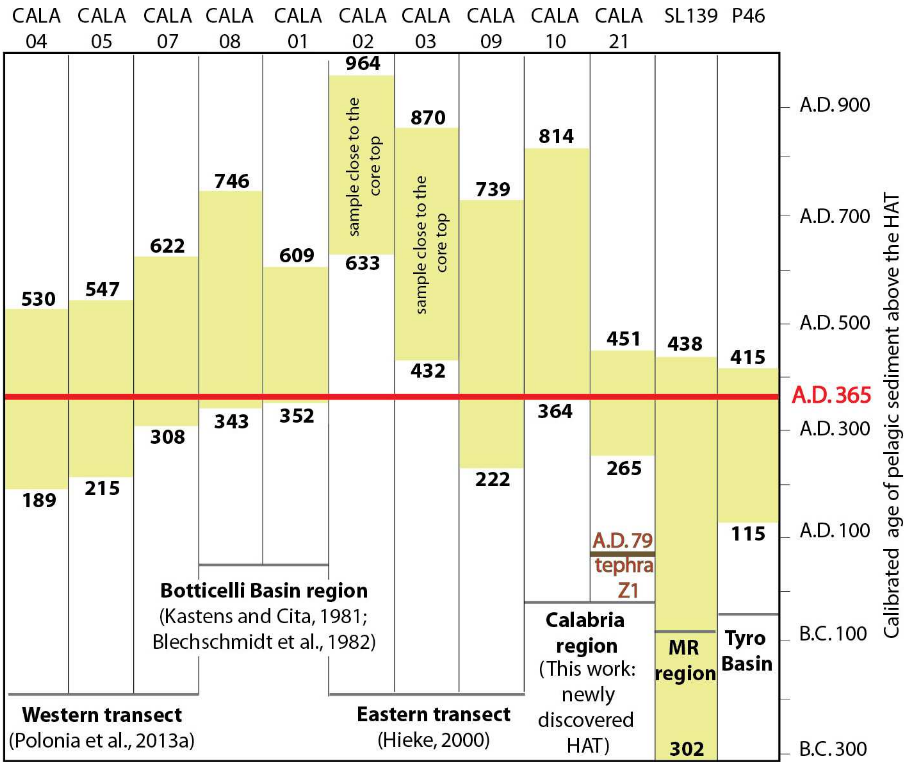

Fig. SM1B *- HAT emplacement time. The dated pelagic samples yield a cluster of ages centered on the AD 365 Crete earthquakes. Where two radiocarbon datings are available for a single sample we used the age obtained with foraminifera. Details for core P46 are given in DR8 and DR9.*

SM2 - Log of core *CALA-07.*

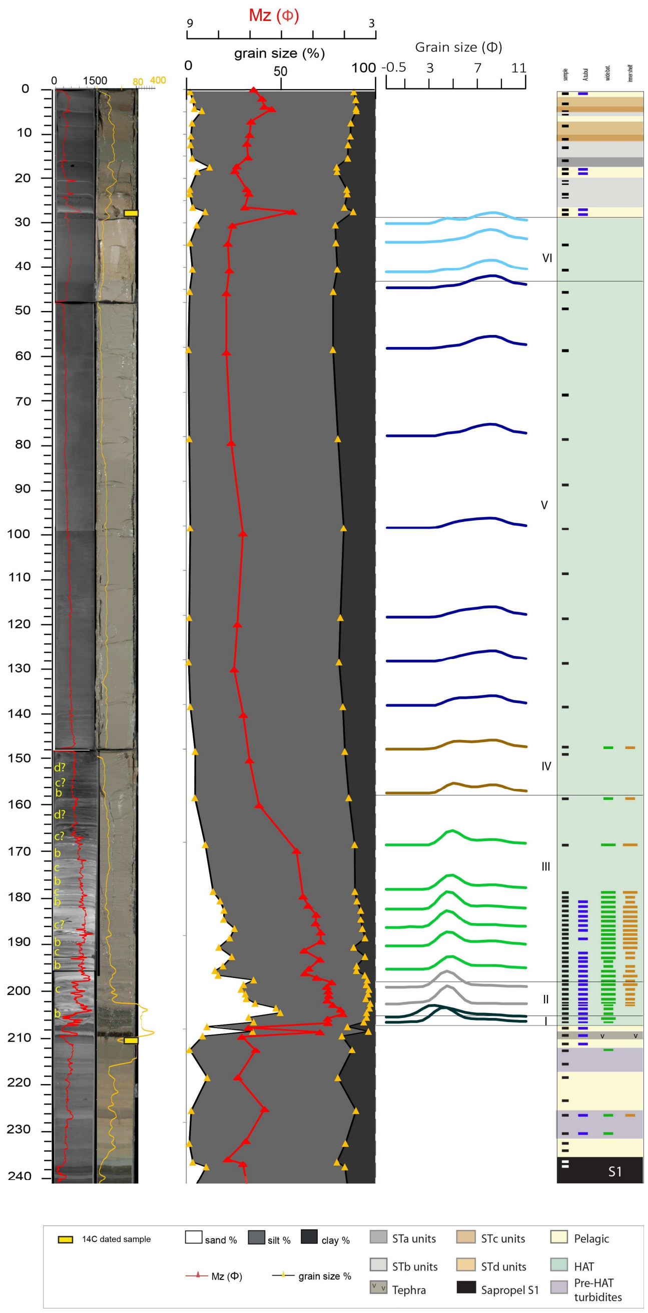

Figure SM2- Log of core *CALA-07.* *From left to right: CAT scan with HU in red color,* photograph with high-resolution magnetic susceptibility in yellow, grain size % with mean diameter in red, grain size modes in different colours for each sediment unit within the HAT, benthic foraminifera assemblages, sedimentary facies for ST1, ST2 and ST3 in different colors as proposed by Polonia et al., (2017), subdivision in individual HAT units (I-VI) in green, pelagic units identified by yellow units while sapropel bed S1 is indicated by black color. Benthic foraminiferal assemblages are grouped in three different classes: blue= abyssal species (mainly *A. tubulosa*); green= wide bathymetric ranges; brown= inner shelf (see Table 2 in the main manuscript for more details). Thin coloured line= rare specimens: < 25 specimens in 0,03 gr of sample; thick coloured line= common specimens: > 25 specimens in 0,03 gr of sample (see Table 2 for details on the bathymetric distribution). Identified Calabrian Arc seismo-turbidites (ST1, ST2 and ST3) with individual units (a, b, c and d) are indicated.

SM3 - Log of core *CALA-08.*

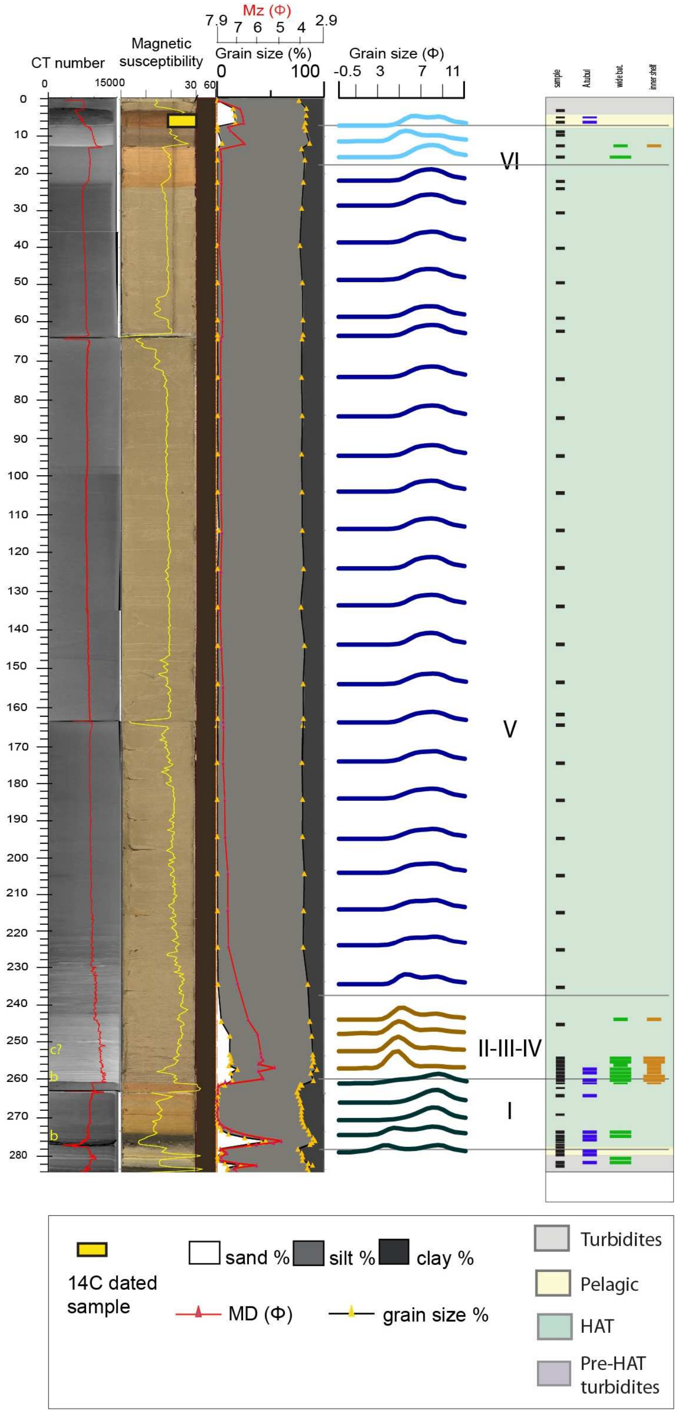

Figure SM3- Log of core *CALA-08.* *From left to right: CAT scan with HU in red color,* photograph with high-resolution magnetic susceptibility in yellow, grain size % with mean diameter in red, grain size modes in different colours for each sediment unit within the HAT, benthic foraminifera assemblages, subdivision in individual HAT units (I-VI) in green and pelagic units identified by yellow units. Benthic foraminiferal assemblages are grouped in three different classes: blue= abyssal species (mainly *A. tubulosa*); green= wide bathymetric ranges; brown= inner shelf (see Table 2 in the main manuscript for more details). Thin coloured line= rare specimens: < 25 specimens in 0,03 gr of sample; thick coloured line= common specimens: > 25 specimens in 0,03 gr of sample (see Table 2 for details on the bathymetric distribution).

SM 4 - Log of core *CALA-21.*

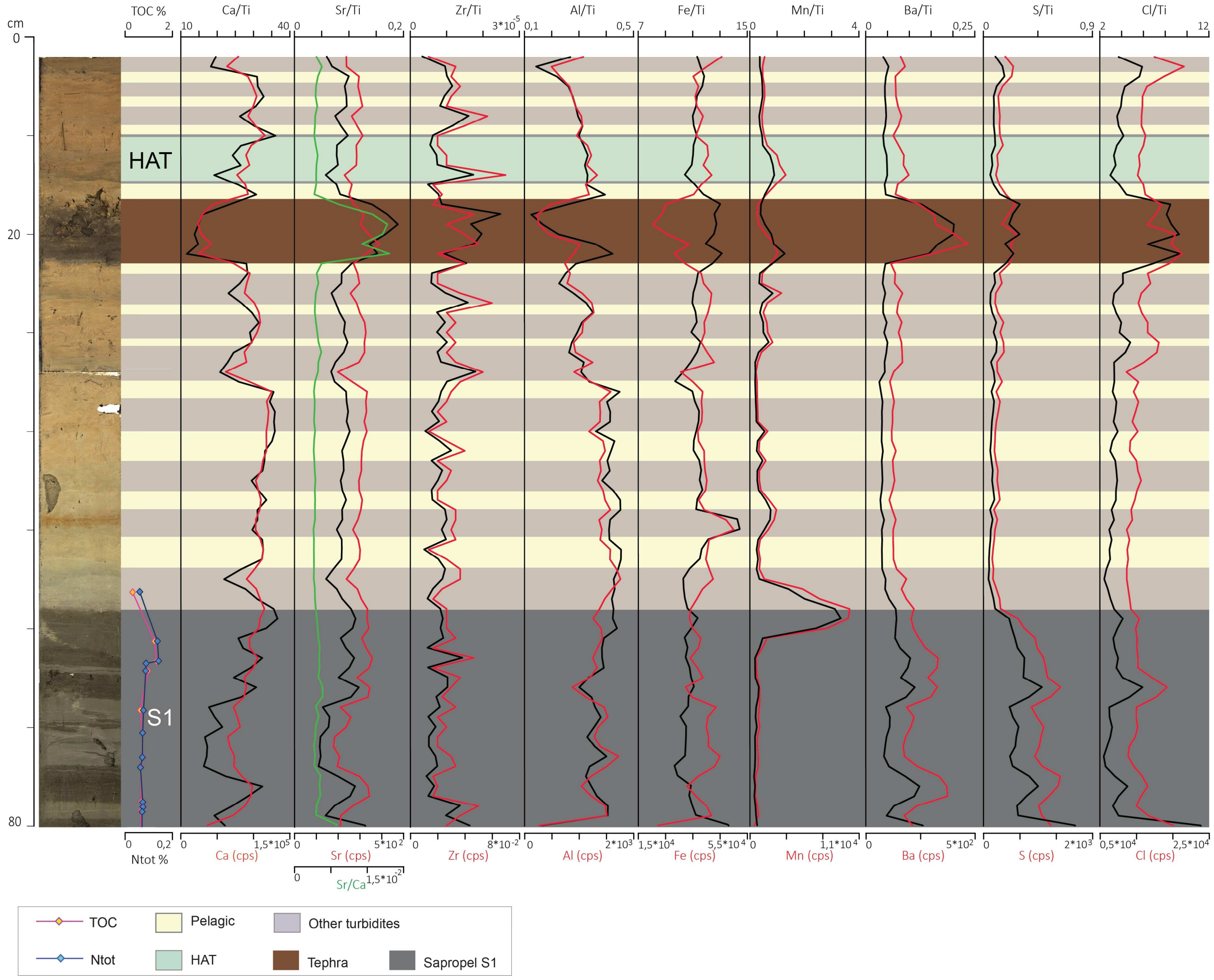

Figure SM4 - Log of core *CALA-21. From left to right:* photograph, total organic carbon (TOC), and XRF data plot along the study sample. Different sedimentary facies for turbidite beds, pelagic units, tephra, HAT and sapropel S1 are indicated in different colors (see figure legend) Modified from Polonia et al. (2015) and Panieri et al. (2013).

SM5 – DESCRIPTION OF THE GRAVITY CORES ANALYSED IN THE MAIN MANUSCRIPT

Abyssal plain setting: core CALA-04 (Fig. 3 in the main manuscript)

The HAT is 1.72 m thick in core CALA-04 and it is located stratigraphically below three terrigenous sandy/silty turbidites (ST1, ST2, ST3 in Fig. 3) that are linked to three major historical Italian earthquakes (i.e. CE 1908, 1693 and 1169; Polonia et al., 2013a; 2017; 2021). Below the HAT, turbidite beds interbedded with cm-thick pelagic units overlie sapropel bed S-1 (Fig. 3).

The HAT base is marked by a sharp increase in sand content (up to 90%) while its top corresponds to a black and red millimetric layer formed by precipitation of Mn and Fe overlain by hemipelagic sediment (Polonia et al., 2013b). The consistent characteristics of the HAT bed are the 40 cm thick sandy base, high values of Ca and Sr elemental concentrations, very high Sr/Ca ratio and the C/N ratio always larger than 10 and up to 19.

The HAT shows a composite structure made up of six different units (Unit-I to -VI) as shown by varying geochemistry and grain size (MD), MS, CT scans, and C/N (Fig. 3). Foraminiferal assemblages are always dominated by planktonic species in association with variable amount of benthic foraminifera, grouped according to their bathymetric distribution (Table 1; Fig. 3).

Unit-I: It is characterized by about 90% of sand with high C/N, which indicate a continental input of sediment (sensu Mayer, 1994). Unit-I is made up of three distinct parallel laminated sub-units that are visible on the CT images as white and black layers more abundant in biogenic and mineral components, respectively. These three sub-units are clearly shown by the MD profile. The sand consists of a heterogeneous mixture of detrital mineral grains (quartz, feldspar, mica - biotite and muscovite, Ca-plagioclase, olivine, augite, carbonate grains, glass shards) and biogenic components mainly given by planktonic foraminifera, with the rare occurrence of reworked species, such as *Globoratalia margaritae*, and subordinate benthic foraminifera represented by taxa indicative of a wide range of bathymetry, from inner shelf to abyssal environments. This unit is characterized by low values of Fe and Zr while it shows a sharp increase in Ca and carbonate relative to the underlying sediment, which suggests a carbonate sediment source and is consistent with the high amount of foraminifera.

Unit-II shows a decrease in sand content to about 70%, reverse grading, detrital mineral grains (quartz, feldspar, mica - biotite and muscovite), benthic foraminifera with a lack of inner shelf foraminifera and a more marine character of C/N. Within this unit, two peaks in MS suggest it is made of two distinct sand stacks. This unit shows a sharp decrease in Ca and an increase in Zr and Fe, indicating a different sediment source relative to Unit-I. It shows parallel bedding at the base and cross bedding upwards in the unit (Tb-Tc Bouma structures).

Unit-III consists of a rather constant sand content (about 75%), CT number and MS, detrital mineral grains (quartz, feldspar, biotite and muscovite) and an increase in C/N up to 18. A large piece of *Posidonia oceanica*, a marine plant living in shallow water depths (0-40 m) is present in this unit that shows an increase in Ca and Sr at its base, while Ca and Sr decrease in the rest of the unit. There are two fining up sequences of MD and Zr and Fe show fluctuations, which suggests a possible stack of two sand sub-units with different composition. It shows parallel bedding at the base and cross bedding upwards in the unit (Tb-Tc Bouma structures). Benthic foraminifera associations include inner shelf taxa.

Unit-IV is characterized by a basal sharp decrease in sand content to 20%, rare mineral grains (quartz, feldspar, mica - biotite and muscovite), a number of sand peaks organized in a saw tooth profile and a sharp increase in Ca and Sr, which remain high up to the HAT top. This unit shows parts of the Bouma Tb to Td structures, and cross bedding on CT images correlates with Zr elemental concentration, which suggests continent-derived sediment inputs as confirmed by the occurrence of abundant inner shelf fauna and high C/N values.

Unit-V is the thickest unit of the turbidite bed and is fining upward, with MD varying from 6 to 7,5. It exhibits some parallel laminations in its central part while the uppermost Unit-V does not show any structure.

C/N is constant at about 12 and lower than the units below. It is barren of benthic foraminifera, whereas small planktonic specimens are locally observed.

Unit-VI is a reddish-yellow bed, which shows faint parallel internal layering on the CT images. It is marked by a sharp decrease in grain size, which shows a saw tooth profile and foraminifera assemblages are comparable with those of Unit-V. The topmost part of the turbidite is pinkish white and enriched in Mn and Fe elemental concentration suggesting the presence of a redox front.

Perched basin, outermost accretionary wedge: core CALA-05 (Fig. 4 in the main manuscript)

The HAT is 1.80 m thick in core CALA-05 and contains a complex depositional sequence that is shown by CT images, grain-size distribution and composition (Fig. 4).

The base of the megabed is defined by a carbonate-rich sandy silt layer, which consists of detrital mineral grains (quartz, feldspar, mica - biotite and muscovite), and biogenic components. The HAT is characterized by geochemical signatures of high Ca, Sr, Ba, S and Cl elemental concentrations, high values of C/N up to 32 and a high sand content up to 70% in the basal units. Similar to CALA-04, foraminiferal assemblages are dominated by planktonic specimens with the secondary occurrences of benthic species with different bathymetric distribution (Tab. 1; Fig. 4). Sedimentology and geochemistry of the core identified 6 major turbidite units (Fig. 4).

Unit-I has a base with up to 25% sand and reverse grading and is truncated by a dark coloured erosive surface of Unit-II (Fig. 4a). Unit-I is marked by a sharp increase in Ca and Sr elemental concentrations and rare detrital mineral grains mainly related to glass shards. The observed benthic foraminifera are indicative of a wide range of bathymetry, from abyssal to inner shelf.

Unit-II is characterized by the highest sand % in the HAT, abundant mineral grains (quartz, feldspar, mica - biotite and muscovite), a basal part with Bouma Tb flat laminations of alternating mm-thick light and dark levels containing abundant biogenic components, mainly planktonic foraminifera and pteropod fragments, and cross lamination units above the laminated base. In some cases, amalgamated intervals of cross-laminated sediments show opposite dipping trends that correlate with a new sand peak. Above the base interval of 1-2 cm, the sequence is normally graded, but with 5 peaks of increased sand concentration, each marked by an increase of Zr and Fe content. A key feature of Unit-II is the low content of carbonate and highest C/N ratios compared to the rest of the HAT. Benthic foraminifera are represented by taxa indicative of a wide range of bathymetry, from abyssal to inner shelf.

Unit-III is marked by a colour change from dark grey of Unit-II to beige, a decrease in sand content with rare mineral grains and a large increase in carbonate content (Fig. 4). Planar lamination is present in the lower part of the layer and possible cross-lamination correlates with a grain-size peak at the top of the layer. Benthic foraminiferal assemblages show species with wide bathymetric distribution, however this unit includes the most abundant amounts of inner shelf species.

The muddy silt Unit-IV is limited at the base by a sharp contact separating the sandy-silt parallel and cross laminated Unit-III from the above Unit-IV depositional sequence. The base of this unit is marked by an increase in Fe, Al, Ba, S and Cl elemental concentrations and shows foraminiferal associations including inner shelf species. Its top is marked by texturally uniform sediments with faint, sand free, laminations.

Unit-V consists of fining upward fine grained sediments associated with a reduction of C/N ratio (still > 10) and a top marked by geochemical anomalies that might be related to a larger water content.

The uppermost Unit-VI shows a saw tooth profile in grain size and is a different reddish beige color marked by faint colour variations. The uppermost part of Unit-VI correlates with geochemical anomalies (Ca, Sr, Fe, Mn). A millimetric black and reddish horizon, enriched in Mn and Fe respectively, is present at the top of the HAT. Units-V and -VI contain a high carbonate content and are almost barren in foraminifera.

Ponded closed basin at a canyon mouth: core CQ14_02 (Fig. 5 in the main manuscript)

The HAT top in core CQ14_02 is 2.62 m below the seafloor suggesting that sediment input is very high in this setting (Fig. 5). Recent sedimentation is related to 5 turbidite beds (ST1, ST1/2, ST2, ST2/2, and ST3) that were deposited during CA historical earthquakes in the last millennia (Polonia et al., 2017). The HAT is 2

m thick and is different relative to the other turbidite beds because of very high C/N values up to 38. Foraminiferal assemblages are dominated by planktonic specimens with subordinate benthic species. The base of the megabed shows a sharp increase in sand content of up to 70% and contains abundant detrital mineral grains (mica – muscovite and biotite - quartz, feldspar) that decrease towards the upper units. Biogenic components consist mainly of *Posidonia oceanica*, pteropod fragments and foraminifera. Geochemical data is more uniform relative to other cores such as CALA-04 and -05, which indicates a more local and/or uniform sediment source. This core does not show the typical increase in Ca like the other cores. It is marked by a slight increase in Sr and a sharp increase of Ba, S and Cl elemental concentrations and a high sand content in the basal unit. Another difference of the HAT at this site is that its grain size is comparable to the most recent turbidite beds while in all other cores HAT grain size is coarser than any other sediment unit. The multiproxy analyses of HAT suggests that in core CQ14_02 it is made up of six units with different geochemical and sedimentological characteristics:

Unit-I (450-462 cm) is a basal massive sand unit (up to 90%) of multiple sub-units with poorly developed Bouma Tb and Tc sedimentary structures and distinct peaks in Zr, Fe and Sr as revealed by XRF data. The most basal laminae (459-462 cm) show an increase in Zr, Ba, S and Cl elemental concentration accompanied by a sharp increase in C/N relative to the pelagic unit below. Benthic foraminifera in the basal sub-unit exhibit a wide range of bathymetric sources but not inner shelf, while shallow marine species are present in the upper stacks.

Unit-II (419-459 cm) is a silty sand (70-80%) that fines upward with two well-developed sets of Bouma b,c sedimentary structures. No distinct sand pulses or geochemical anomalies are present within this unit, which shows rather constant trends. Benthic foraminifera assemblages are indicative of a wide range of bathymetric sources including inner shelf taxa.

Unit-III (361-419 cm) is a silty sand unit with a sand peak (70%) and Tc Bouma sedimentary structure at the base. Unit-III is marked by a large increase in C/N up to 38, which is mainly related to an increase in C_{org}. This peak is also associated with geochemical anomalies possibly related to an increase in water content (increase in Cl elemental concentration). A second sand peak and increase in C/N ratio occurs at the top of the unit. Benthic foraminiferal assemblages show a wide range of bathymetric sources including the inner shelf.

Unit-IV (316-361 cm) exhibits a fining upward trend with sub-units (354-365 cm, 320-340 cm) that could be related to disturbances introduced during coring operations. However, their association with geochemical anomalies suggests that they might represent a new sediment input. Foraminiferal assemblages show a wide range of bathymetric sources including inner shelf fauna in the convoluted sub-unit but not at the base of Unit-IV.

Unit-V (276-316 cm): this homogenous muddy silt unit exhibits few internal structures in CT images, but faint planar bedding is found at its base while faint possible convoluted layers occur in the topmost part of the unit where C/N is < 10. Foraminiferal assemblages are characterized by few small-sized planktonic specimens, while rare benthic foraminifera are present at the base of the unit.

Unit-VI (260-276 cm) exhibits a sharp increase in $CaCO_3$, Sr and Ca accompanied by high values of Fe and a pronounced peak in Mn at its top corresponding to the redox front. Unit-VI contains white and olive grey cm-thick undulating laminae, which were studied with high resolution (1 mm spacing) of XRF data and OC (Fig. 5). White laminae are high in Sr, Ca, Mn and low in Fe while olive-grey laminae are high in Fe, Al, Ba and low in Sr, Ca and Mn. These variations are accompanied by variations in C/N, which is < 10 but higher in the dark laminae. The unit includes only rare and small-sized planktonic foraminifera.

Ponded isolated basins not fed by canyons: core CALA-01 (Fig. 6 in the main manuscript)

The HAT in core CALA-01 is 3.48 m thick and the thickest HAT bed in our cores (Fig. 6). Its top is covered by 20 cm of more recent sediments represented by an alternation of pelagic units and thin turbidite beds. The base of the HAT is located about 18 cm above the top of sapropel S1. The HAT is characterized by a sharp increase in sand content up to 65% containing mainly biogenic components (foraminifera, pteropods, vegetal remains), and very rare detrital mineral grains (quartz, feldspars and mica). For this core, only grain size and micropaleontological analyses are available and they distinguish only four main units.

Unit I (362-366 cm) consists of a sharp sand base with faint parallel lamination and then fines upward to a sandy silt. Foraminiferal assemblages are dominated by planktonic taxa with the occurrence of rare benthic species indicative of a wide range of bathymetry, excluding the inner shelf.

Units-II to-IV (329-362 cm) is a silt layer that correlates with abundant inner shelf species in the benthic foraminiferal assemblages.

Unit V (26-329 cm) is a light grey clayey silt unit with little sand, which shows a rather homogeneous structure with no internal layering or disturbances. The majority of samples include only rare and small planktonic foraminifera.

Unit-VI (20-24 cm) is the topmost brown unit marked by a sharp base on X-ray, and foraminifera assemblages characterized by several planktonic specimens and rare benthic taxa indicative of a wide range of bathymetry. The top of the turbidite is marked by a millimetric thick red layer representing the redox front.

SM6 - Supplementary 6 - taxonomic reference list

This list includes foraminiferal *taxa* cited in the text and supplementary

Ammonia beccarii - Nautilus beccarii Linnaeus 1758, p. 710.

Articulina tubulosa - Quinqueloculina tubulosa Seguenza, 1862, p. 129, pl. 2, figs. 8, 8a, 8b.

Asterigerinata mamilla - Rotalina mamilla Williamson, 1858, p. 54, pl. 4, figs. 109-111.

Biasterigerina planorbis - Asterigerina planorbis d'Orbigny, 1846, p. 205, pl. 11, figs. 1-3.

Bolivina - Bolivina d'Orbigny, 1839b, p. 60.

Bolivina alata - Vulvulina alata Seguenza, 1862, p. 125, pl. 2, figs. 5-5a.

Bolivina variabilis - Textularia variabilis Williamson, 1858, p. 76, pl. 6, figs. 162-163.

Bulimina - Bulimina d'Orbigny, 1826, p. 269.

Bulimina aculeata - Bulimina aculeata d'Orbigny, 1826, p. 269.

Bulimina marginata - Bulimina marginata d'Orbigny, 1826, p. 269, pl. 12, figs. 10-12.

Cassidulina carinata - Cassidulina laevigata var. *carinata* Silvestri, 1896, p. 104, pl. 2, figs. 10a-c.

Cibicidoides - Cibicidoides Thalmann, 1939, p. 448.

Cibicidoides pseudoungerianus - Truncatulina pseudoungeriana Cushman, 1922a, p. 69, pl. 24, fig. 1.

Cibicidoides ungerianus - Rotalina ungeriana d'Orbigny, 1846, p. 157, pl. 8, figs. 16-18.

Elphidium - Elphidium Montfort, 1808, p. 15.

Elphidium advenum - Polystomella advena Cushman, 1922b, p. 56, pl. 9, fgs. 11-12

Elphidium macellum - Nautilus macellus Fichtel & Moll, 1798, p. 68, pl. 10, figs. e, g, h, k.

Fissurina - Fissurina Reuss, 1850, p. 366.

Fissurina marginata - Vermiculum marginatum Montagu, 1803, p. 2, pl. 1, fig. 7.

Gavelinopsis praegeri - Discorbina praegeri Heron-Allen & Earland, 1913, p. 122, pl. 10, figs. 8-10.

Globobulimina affinis - Bulimina affinis d'Orbigny, 1839a, p. 105, pl. 2, figs. 25-26.

Globocassidulina subglobosa - C*assidulina subglobosa* Brady, 1881, p. 60, pl. 54, figs. 17a-c.

Gyroidina - *Gyroidina* d'Orbigny, 1826, p. 278.

Gyroidina altiformis - *Gyroidina soldanii* var. *altiformis* Stewart & Stewart, 1930, p. 67, pl. 9, figs. 2a-c.

Gyroidinoides - *Gyroidinoides* Brotzen, 1942, p. 19.

Lagena - *Lagena* Walker & Jacob, 1798, p. 634.

Lagena striata - *Oolina striata* d'Orbigny, 1839b, p. 21, pl. 5, fig. 12.

Lagena substriata - *Lagena substriata* Williamson, 1848, p. 15, pl. 2, fig. 12.

Lobatula lobatula - *Nautilus lobatulus* Walker & Jacob, 1798, p. 642, pl. 14, fig. 36.

Melonis affinis - *Nonionina affinis* Reuss, 1851, p. 72, pl. 5, figs. 32a-b.

Nonionoides turgidus - *Rotalina turgida* Williamson, 1858, p. 50, pl. 4, figs. 95-97.

Porosononion - *Porosononion* Putrya in Voloshinova, 1958, p. 125.

Pyrgo - *Pyrgo* Defrance, 1824, p. 273.

Pyrgo lucernula - *Biloculina lucernula* Schwager, 1866, p. 202, pl. 4, figs. 17a-b.

Quinqueloculina seminulum - *Serpula seminulum* Linnaeus, 1758, p. 786.

Quinqueloculina - *Quinqueloculina* d'Orbigny, 1826, p. 301.

Quinqueloculina bosciana - *Quinqueloculina bosciana* d'Orbigny, 1839a, p, 191, pl. 11, figs. 22-24.

Reussella spinulosa - *Verneuilina spinulosa* Reuss, 1850, p. 374, pl. 47, figs. 12a-c.

Rosalina - *Rosalina* d'Orbigny, 1826, p. 271.

Rosalina bradyi - *Discorbis globularis* var. *bradyi* Cushman, 1915, p. 12, pl. 8, figs. 1a-c.

Spirillina vivipara - *Spirillina vivipara* Ehrenberg, 1843, p. 323, 422, pl. 3, fig. 41.

Trifarina angulosa - *Uvigerina angulosa* Williamson, 1858, p. 67, pl. 5, fig. 140.

Uvigerina - *Uvigerina* d'Orbigny, 1826, p. 268.

Uvigerina mediterranea - *Uvigerina mediterranea* Hofker, 1932, p. 118, figs. 32a-g.

Valvulineria bradyana - *Discorbina bradyana* Fornasini, 1900, p. 393, fig. 43.

SM7 - Photomicrographs of selected benthic foraminifera in core samples

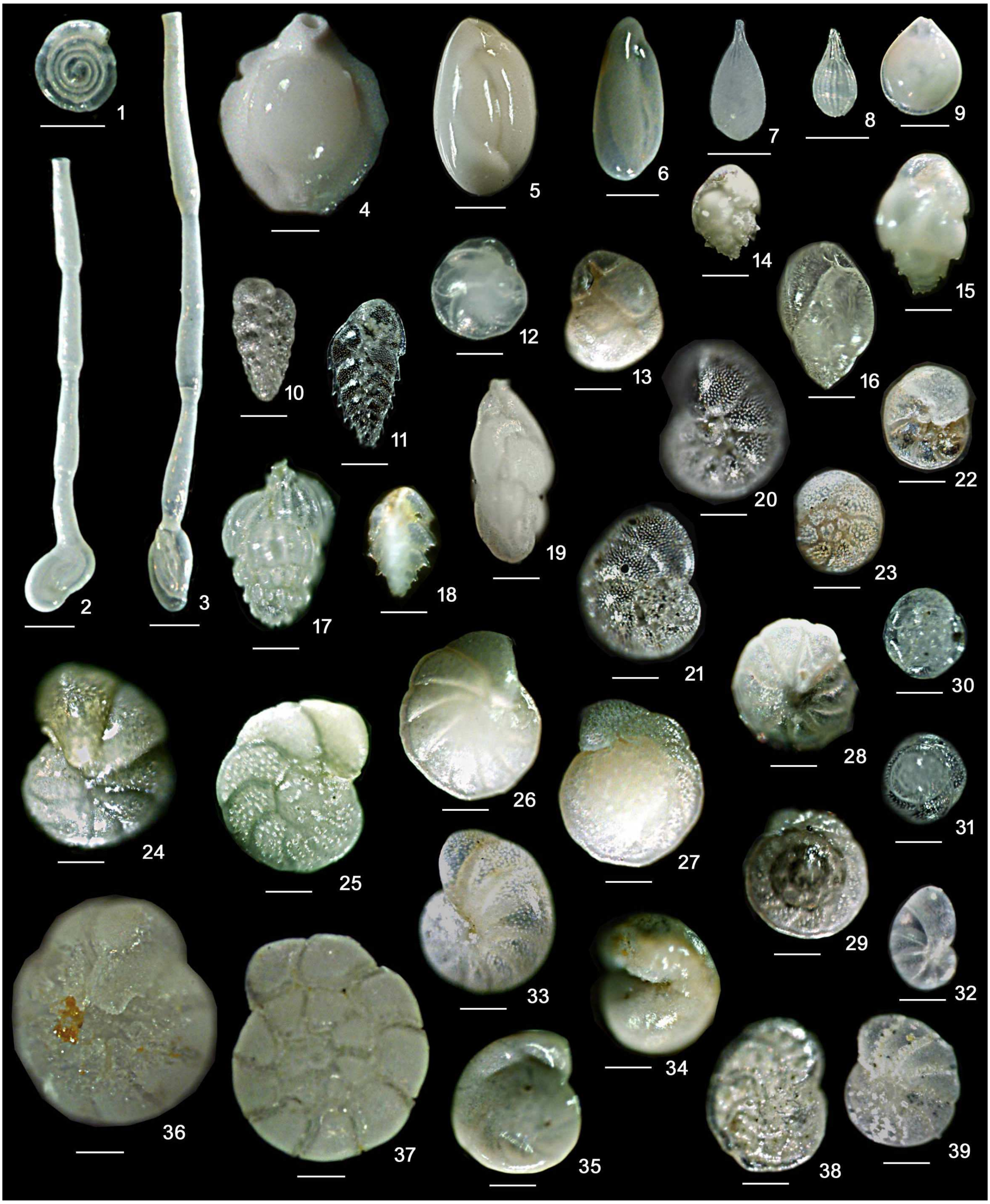

Plate 1 - 1 *Spirillina vivipara* (core CALA-04 cm 184). 2-3 *Articulina tubulosa* (core CQ14-02 cm 464). 4 *Pyrgo lucernula* (core CQ14-02 cm 460). 5 *Quinqueloculina seminula* (core CALA-04 cm 246). 6 *Quinqueloculina bosciana* (core CQ14-02 cm 403). 7 *Lagena substriata* (core CQ14-02 cm 403). 8 *Lagena striata* (core CQ14-02 cm 403). 9 *Fissurina marginata* (core CQ14-02 cm 460). 10 *Bolivina variabilis* (core CQ14-02 cm 460). 11 *Bolivina alata* (core CQ14-02 cm 403). 12 *Cassidulina carinata* (core CALA-04 cm 184). 13 *Globocassidulina subglobosa* (core CALA-04 cm 163). 14 *Bulimina aculeata* (core CQ14-02 cm 460). 15 *Bulimina marginata* (core CALA-04 cm 163). 16 *Globobulimina affinis* (core CALA-04 cm 246). 17 *Uvigerina mediterranea* (core CQ14-02 cm 403). 18 *Reussella spinulosa* (core CQ14-02 cm 403). 19 *Trifarina angulosa* (core CALA-04 cm 246). 20-21 *Valvulineria bradyana* (core CQ14-02 cm 403), 20 umbilical view; 20 spiral view. 22-23 *Rosalina bradyi* (core CALA-04 cm 246), 22 umbilical view; 23 spiral view. 24-25 *Lobatula lobatula* (core CALA-04 cm 246), 24 umbilical view; 25 spiral view. 26-27 *Cibicidoides pseudoungerianus* (core CQ14-02 cm 460), 26 umbilical view; 27 spiral view. 28-29 *Cibicidoides ungerianus* (core CQ14-02 cm 460), 28 umbilical view; 29 spiral view. 30-31 *Asterigerinata mamilla* (core CALA-04 cm 246), 30 umbilical view; 31 spiral view. 32 *Nonionella turgida* (core CQ14-02 cm 403). 33 *Melonis affinis* (core CALA-04 cm 163). 34-35 *Gyroidina altiformis* (core CALA-04 cm 163), 34 umbilical view; 35 spiral view. 36-37 *Ammonia beccarii* (core CALA-04 cm 246), 36 umbilical view; 37 spiral view. 38 *Elphidium macellum* (core CALA-04 cm 246). 39 *Elphidium advenum* (core CQ14-02 cm 460). Bar: 100 µm.

SM8 – Sedimentological and geochemical evidences of laminites in Unit-VI

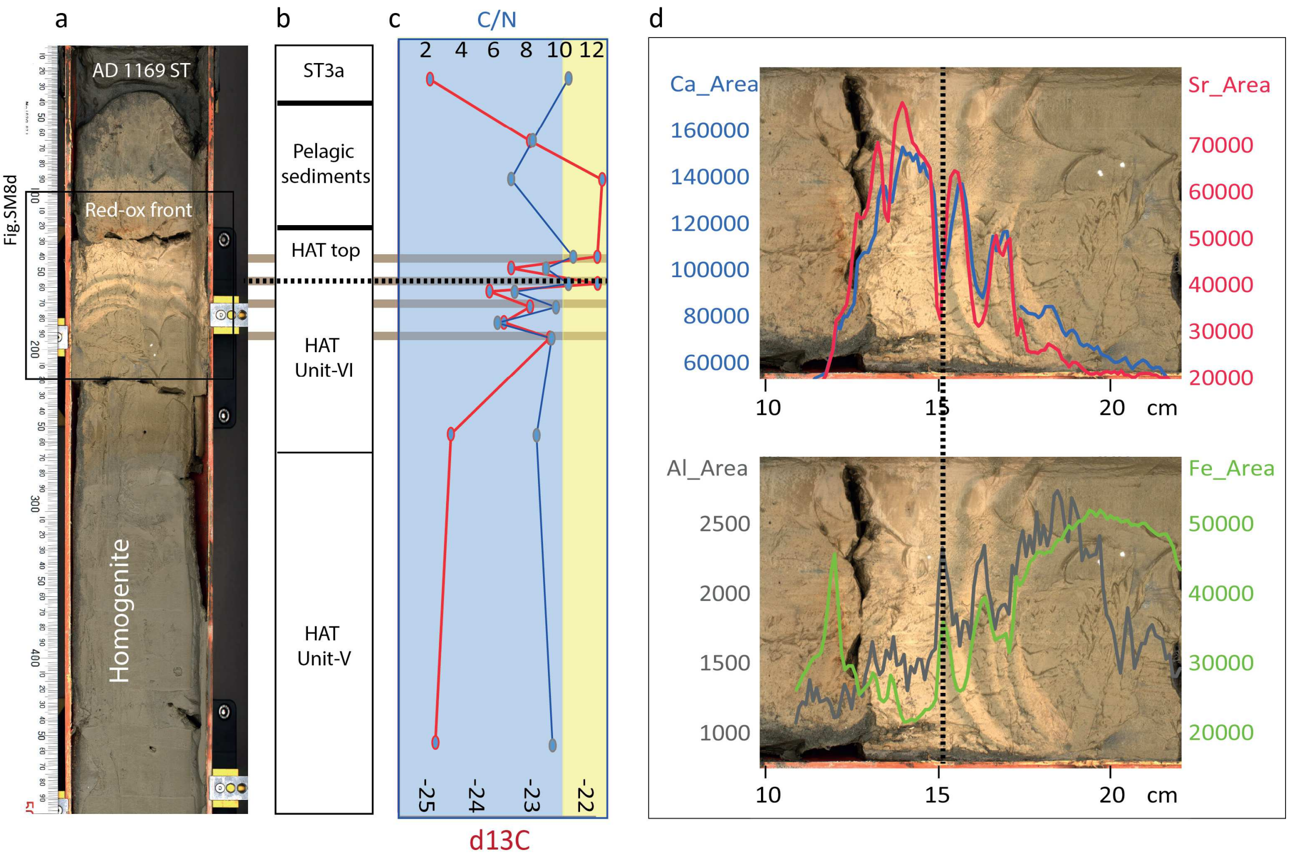

SM8 – a) Photograph of topmost part of HAT in core CQ14_02. b) Sediment units within the HAT (Unit-V homogenite and Unit-VI Seiche), the redox layer ontop the HAT, pelagic sediments deposited when the ecosystem resumed after the catastrophic event and ST3 whose deposition was triggered by the CE 1169 Sicily earthquake. c) organic carbon data (C/N in blue and $\delta^{13}C_{org}$ in red. Blue areas if C/N<10, yellow areas if C/N>10). XRF data plot along the study sample shown in Fig. 9A: Sr (red), Ca (blue), Fe (green) and Al (grey). Variation in organic carbon and XRF data highlight the different composition of the dark and light laminae constituting Unit-VI. Dark lines in Fig. 9c represent dark laminae. The dark laminae at about 15 cm is marked as a reference levels in Figs. 9c and 9d.

For the historic CE 1908 Messina earthquake, eyewitnesses reported a rough sea, which lasted one day or more after the initial tsunami wave. These post-tsunami water mass movements were described as decreasing oscillations with a period of 12-14 minutes and amplitudes of 5-6 m lasting for 5 hours after the main tsunami wave (Baratta, 1910). In the confined basin of Lake Lucerne Switzerland, the shallow Lucerne Bay during the 1601 CE earthquake and tsunami, historical chronicles report a seiche with an amplitude of about 1 to 2 m and an initial period of 10 minutes. Its amplitude decreased with time but the seiche persisted for several days after the event (Kremer et al., 2015). These observations support our interpretation that Unit-VI is related to seiching oscillations in the confined basin of the Ionian Sea during the CE 365 earthquake and tsunami.

SM9 – Linear discriminant analysis (LDA) based on XRF-CS data

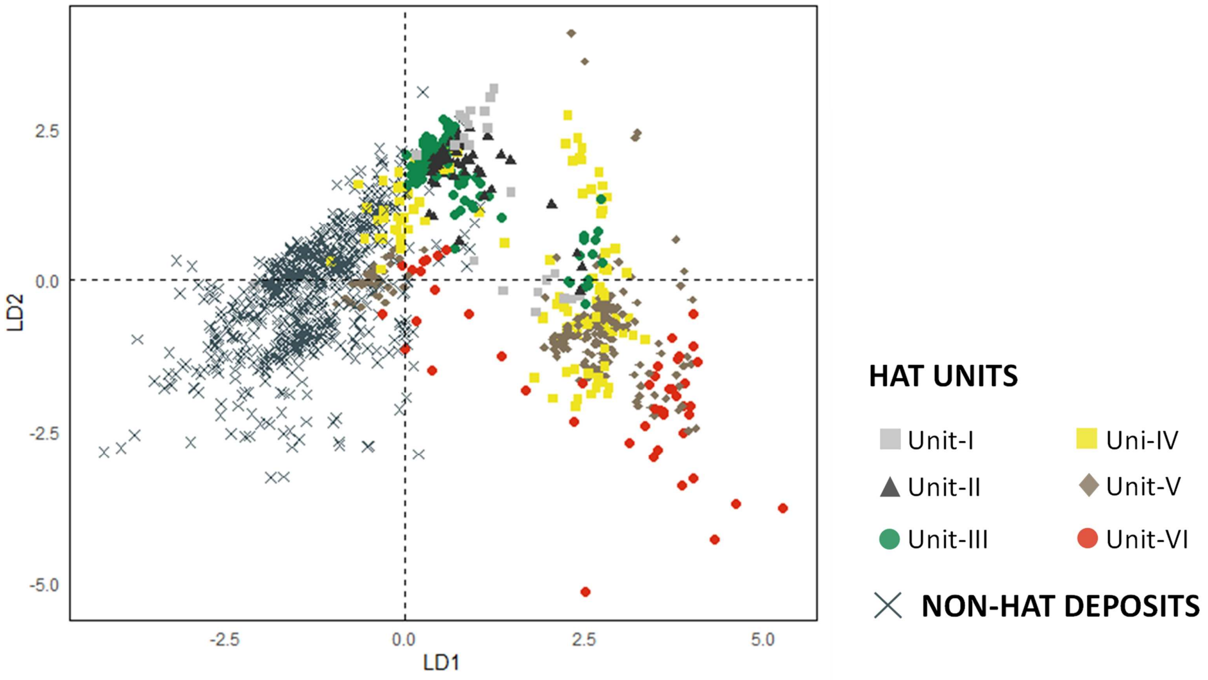

SM9 - Linear discriminant analysis (LDA) based on XRF-CS datasets for the cores CALA-04, CALA-05 and CQ14_02 (analyzed together, n=1225). Score plot discriminating the non-HAT deposits and the six HAT units.

SM10 - Photographs of a selected fragment of Posidonia oceanica in core CALA-04

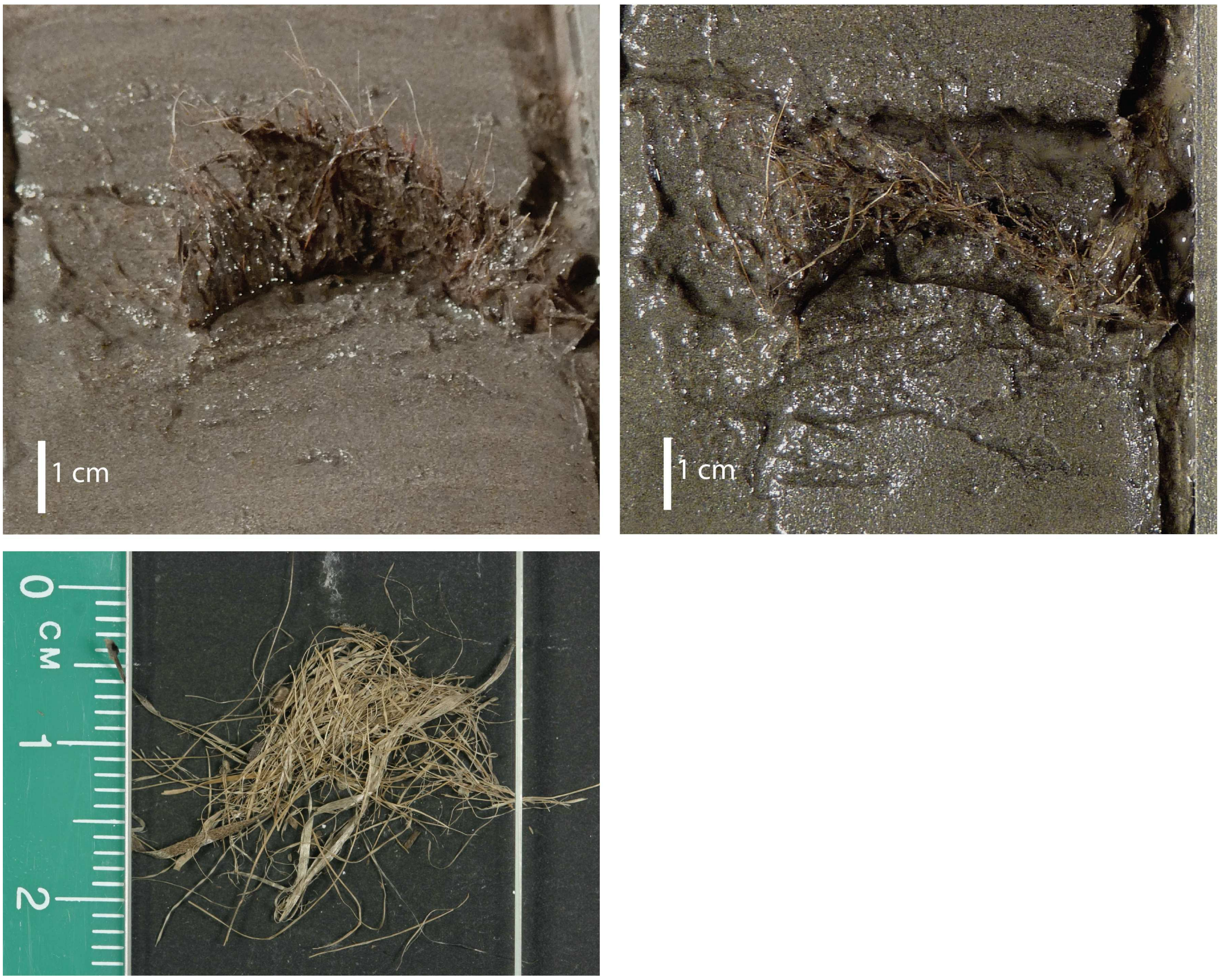

SM11 – PGA %g of M_w>8 earthquakes occurred worldwide after 2000 with a similar ipocentral depth to the Crete earthquake.

EARTHQUAKE MAGNITUDE	DATE	LOCATION	DEPTH (Km)	PGA %g USGS
8,1	2021-08-12	South Sandwich	55,7	0,2-0,5
8,2	2021-07-29	Alaska	35,0	0,5-1,0
8,1	2021-03-04	Kermadec Islands	28,9	0,5-1.0
8,2	2017-09-08	Mexico	47,4	0,2-0,5
8,3	2015-09-16	Illapel, Chile	22,4	0,5-1,0
8,2	2014-04-01	Iquique, Chile	25,0	0,2-0,5
8,0	2013-02-06	Solomon Islands	24,0	< 0,1
8,2	2012-04-11	Northern Sumatra	25,1	0,2-0,5
8,6	2012-04-11	Northern Sumatra	20,0	0,2-0,5
8,8	2010-02-27	Chile	22,9	0,5-1,0
8,4	2007-09-12	Indonesia	34,0	~ 0,2
8,0	2007-08-15	Peru	39,0	0,2-0,5
8,1	2007-04-01	Solomon Islands	24,0	0,2-0,5
8,0	2006-05-03	Tonga	55,0	~ 0,5
8,6	2005-03-28	Indonesia	30,0	~ 1,0
8,2	2003-09-25	Japan	27,0	~ 0,5
8,4	2001-06-23	Peru	33,0	0,5-1.0
8,0	2000-11-16	Papua New Guinea	33,0	0,2-0,5

Table SM11 – Depth and PGA of M>8 earthquakes occurred worldwide after 2000 with a similar ipocentral depth to the Crete earthquake. USGS data (https://earthquake.usgs.gov/earthquakes) 27 Sept, 2021.

SM 12 -Polygon area distribution for the estimate of HAT sediment budget

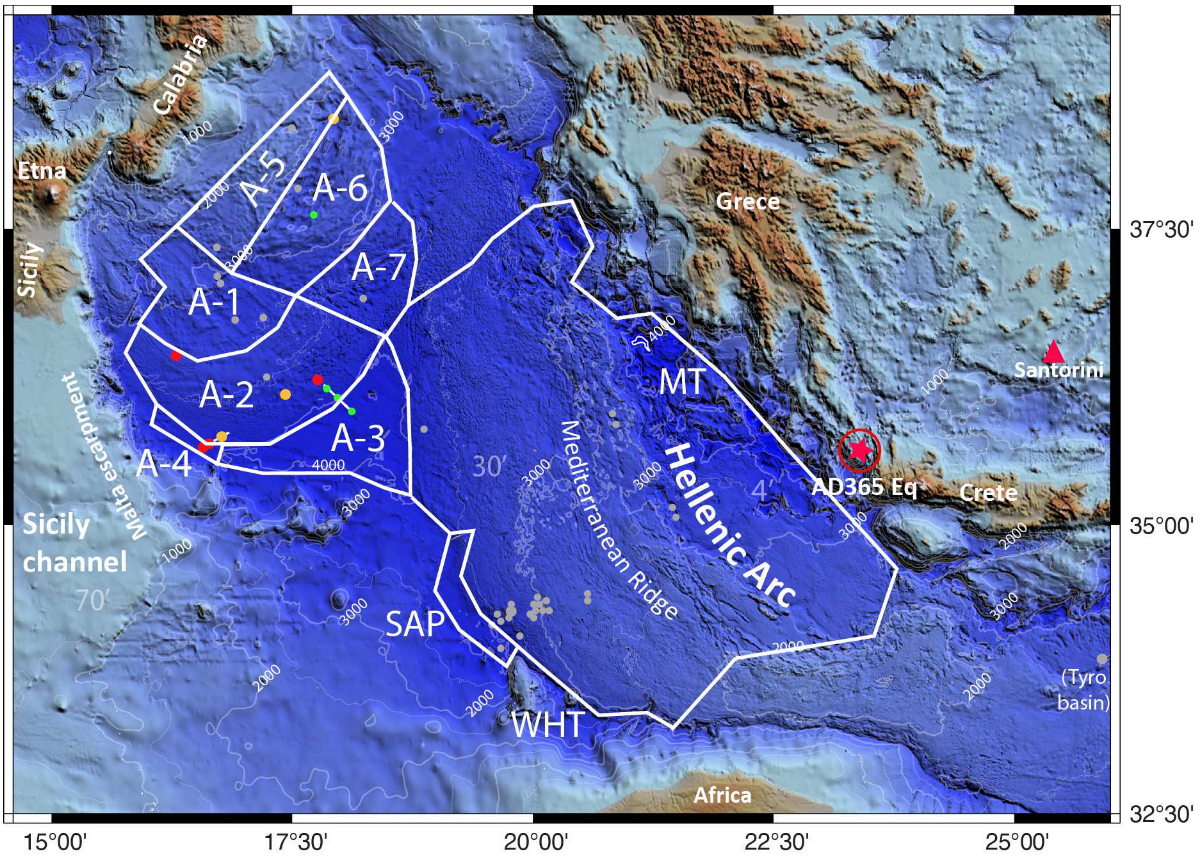

Fig. SM12 - Morphobathymetric data are from Global Bathymetry and Elevation Digital Elevation Model: SRTM30_PLUS v8 (https ://data.gov.au/data/datas et/globa l-hills hadin g-from-srtm3 0_plus-v8-0-nerp-te-13-1-eatla s-source-ucsd/ - Becker et al., 2009). The map was compiled using GMT package (version 6.0.0; https ://www.gener ic-mappi ng-tools .org/), and the image was edited using Adobe Illustrator (CS6; https ://www.adobe .com/).

Polygon area distribution for the estimate of HAT sediment budget

We divided the depositional area in the Calabrian Arc into seven polygons with rather homogenous seafloor physiography and HAT thickness. For each of these areas we have utilized a HAT characteristic thickness derived from the analyses of the reference cores.

Area-1: relatively flat area fed by canyon systems in the Messina Straits region (reference core CQ14_02 in Fig. 5 of the main manuscript, HAT thickness = 2 m). This area is about $14x10^3 km^2$ but contains at least 60% of slopes where the HAT did not deposit or has a negligible thickness. Considering this data, the total volume of HAT in Area-1 is about 20 km^3.

Area-2: Cobblestone area of the western lobe of the Calabrian Arc accretionary wedge (reference cores: CALA-01, -08, -02, -03 Figs. 6 and 7 of the main manuscript; average HAT thickness = 2,5 m). This area is about $15x10^3 km^2$ but contains at least 60% of slopes where the HAT has negligible thickness. Considering this data, the total volume of HAT in Area-2 is about 15 km^3.

Area-3: Ionian abyssal plain. Reference core CALA-09 in Fig. 7 of the main manuscript. This core did not sample the HAT base and we refer to Chirp profiles in Fig. 2 where the HAT is 12 m thick combined with previous reconstructions made by Rebesco et al., 2000 (23 m thickness). From these data we estimate an average thickness of about 20 m. This polygon has an area of about $10x10^3 km^2$ and thus the total volume of HAT in Area-3 is about 200 km^3.

Area-4: Ionian abyssal plain margins. Reference core: CALA-04 (Figs. 3 and 7 of the main manuscript). Average HAT thickness = 2 m. The area is about 1.5 $x10^3 km^2$ providing a total volume of about 5 km^3.

Area-5: Inner plateau of the Eastern lobe of the CA accretionary wedge. Reference cores: CALA-20 (Fig. 7 of the main manuscript) with an average HAT thickness = 2.6 m. The area is about $10x10^3 km^2$ but contains about 50% of bathymetric slopes with negligible HAT thickness and contribute a total volume of about 13 km^3.

Area-6: Inner wedge of the CA eastern lobe reference cores: CALA-10 and -15 (Fig. 7 of the main manuscript) with an average HAT thickness = 2 m. The area is about $13x10^3 km^2$ but it contains about 60% of bathymetric slopes with negligible HAT thickness and thus contributes a total volume of about 5 km^3.

Area-7: Outer wedge, cobblestone topography of the CA eastern lobe reference cores: CQ14_01 with an average HAT thickness = 8 m. The area is about 8 $x10^3 km^2$ but contains about 50% of bathymetric slopes with negligible HAT thickness providing a total volume of about 4,0 km^3.

From these data, the total volume of the HAT in the Ionian Sea is estimated to be about 200 km^3.

References

Baratta, M. (1910). La catastrofe sismica calabro-messinese (28 Dicembre 1908). Relazione alla Società Geografica Italiana. Roma, 2 voll. (Text and Atlas), pp. XV, 426 *(*OCLC 38646170*;* https://www.worldcat.org/title/terremoto-calabro-siculo-del-28-dicembre-1908-messina/oclc/38646170*).*

Becker, J. J., Sandwell, D. T., Smith, W. H. F., Braud, J., Binder, B., Depner, J., Fabre, D., Factor, J., Ingalls, S., Kim, S-H., Ladner, R., Marks, K., Nelson, S., Pharaoh, A., Trimmer, R., Von Rosenberg, J., Wallace, G. and Weatherall, P.(2009)'Global Bathymetry and Elevation Data at 30 Arc Seconds Resolution: SRTM30_PLUS',Marine Geodesy,32:4,355 — 371

Brady, H. B. (1881). Notes on some of the Reticularian Rhizopoda of the "Challenger" Expedition. Part III. Quarterly Journal of Microscopical Science. n.s. 21, 31-71.

Bronk Ramsey, C. (2008). Deposition models for chronological records. Quaternary Science Reviews 27, 42-60.

Brotzen, F. (1942). Die Foraminiferengattung Gavelinella nov. gen. und die Systematik der Rotaliiformes. *Sveriges Geologiska Undersökning* ser. C*,* no. 451, 1-60.

Cushman, J. A. (1915). A monograph of the Foraminifera of the North Pacific Ocean. Part V. Rotaliidae. Bulletin of the United States National Museum, 71, 1-87.

Cushman, J. A. (1922a). The foraminifera of the Byram Calcareous Marl at Byram, Mississippi. United States Geological Survey Professional Papers, 129, 87-123.
Cushman, J. A. (1922b). Shallow-water Foraminifera of the Tortugas region. Publications of the Carnegie Institution of Washington 311. Department of Marine Biology of the Carnegie Institution of Washington, 17, 1-85.
Defrance, J.L.M. (1824). Mollusques, vers et zoophytes. *In* Dictionnaire Des Sciences Naturales. Paris, France, 32, 177 p.
Ehrenberg, C.G. (1843). Verbreitung und Einfluss des mikroskopischen Lebens in Süd-und Nord-Amerika. Abhandlungen der Königlichen Akademie der Wissenschaften zu Berlin, 291-445.
Fichtel, L.; Moll, J.P.C. (1798). Testacea microscopia, aliaque minuta ex generibus Argonauta et Nautilus, ad naturam delineata et descripta. A. Pichler, Wien. 123 p.
Fornasini, C. (1900). Intorno ad alcuni esemplari di foraminiferi adriatici. *Memorie della Reale Accademia delle Scienze dell'Istituto di Bologna,.* ser. 5, t. 8, 357-402.
Heron-Allen, E., Earland, A. (1913). Clare Island Survey: Part 64. Foraminifera. Proceedings of the Royal Irish Academy*.* 31 sect. 3, 188 p.
Hofker, J. (1932). Notizen über die Foraminiferen des Golfes von Neapel, III. Die Foraminiferenfauna der Ammontatura. Pubblicazioni Stazione Zoologica di Napoli*,* 12, 61-144.
Kremer, K., Hilbe, M., Simpson, G., Decrouy, L., Wildi, W., Girardclos, S. (2015). Reconstructing 4000 years of mass movement and tsunami history in a deep peri-Alpine lake (Lake Geneva, France-Switzerland). Sedimentology 62(5), 1305-1327. doi.org/10.1111/sed.12190
Linnaeus, C. (1758). *Systema Naturae*. 10th ed., Holmiae. Stockholm. 824 p.
Mayer, L.M. (1994). Surface area control of organic carbon accumulation in continental shelf sediments. Geochimica et Cosmochimica Acta 58(4), 1271-1284
Montagu, G. (1803). Testacea Britannica or natural history of British shells, marine, land, and fresh-water, including the most minute: Systematically arranged and embellished with figures. J. White, London, 1-606.
Montfort, P. (Denis de) (1808). Conchyliologie systématique et classification méthodique des coquilles. Paris: Schoell*.* 1, 409 p.
Orbigny, A. D. d'. (1826). Tableau méthodique de la classe des Céphalopodes. *Annales des Sciences Naturelles.* vol. 7: 96-169, 245-314.
Orbigny, A. D. d'. (1839b). Voyage dans l'Amérique Méridionale. Foraminifères. t. 5 pt. 5, 1-86.
Orbigny, A. D. d'. (1839a). Foraminifères, in de la Sagra R., Histoire physique, politique et naturelle de l'ile de Cuba. *A. Bertrand.* 224 p.
Orbigny, A. D. d'. (1846). Die fossilen Foraminiferen des tertiären Beckens von Wien. Foraminifères fossiles du bassin tertiaire de Vienne. 312 p..
Panieri, G., Polonia, A., Lucchi, R.G., Zironi, S., Capotondi, L., Negri, A., Torelli, L. (2013). Mud volcanoes along the inner deformation front of the Calabrian Arc accretionary wedge (Ionian Sea). Marine Geology 336, 84-98. https://doi.org/10.1016/j.margeo.2012.11.003.
Polonia, A., Panieri, G., Gasperini, L., Gasparotto, G., Bellucci, L.G., Torelli, L. (2013a). Turbidite paleoseismology in the Calabrian Arc subduction complex (Ionian Sea). Geochem. Geophys. Geosyst. 14, 112–140. http://dx.doi.org/10.1029/2012GC004402.
Polonia, A., Bonatti, E., Camerlenghi, A., Lucchi, R.G., Panieri, G., Gasperini, L. (2013b). Mediterranean megaturbidite triggered by the AD 365 Crete earthquake and tsunami. Sci. Rep. 3, 1285. http://dx.doi.org/10.1038/srep01285
Polonia, A., Romano, S., Çağatay, N.M., Capotondi, L., Gasparotto, G., Gasperini, L., Panieri, G., Torelli, L. (2015). Is repetitive slumping during sapropel S1 related to paleo-earthquakes? Marine Geology 361, 41–52.
Polonia, A., Vaiani, C.S., De Lange, G. (2016). Did the AD 365 Crete earthquake / tsunami trigger synchronous giant turbidity currents in the Mediterranean Sea? Geology 44, 191–194. http://dx.doi.org/10.1130/G37486.1.
Polonia, A., Nelson, C.H., Romano, S., Vaiani, S.C., Colizza, E., Gasparotto, G., Gasperini, L. (2017). A depositional model for seismo-turbidites in confined basins based onIonian Sea deposits. Marine Geology 384, 177–198. Doi: https://doi.org/10.1016/j.margeo.2016.05.010.

Polonia A., Bonetti, C., Bonetti, J., Çağatay, M.N., Gallerani, A., Gasperini, L., Nelson, C.H., Romano, S. (2021). Deciphering co-seismic sedimentary processes in the Mediterranean Sea using elemental, organic carbon and isotopic data. Geochemistry, Geophysics, Geosystems. https://doi.org/10.1029/2020GC009446.

Rebesco, M., Della Vedova, B., Cernobori, L., Aloisi, G. (2000). Acoustic facies of Holocene megaturbidites in the Eastern Mediterranean. Sedimentary Geology 135, 65–74.

Reimer, P. J., Baillie, M. G. L., Bard, E., Bayliss, A., Beck, J. W., Blackwell, P. G., Bronk Ramsey, C., Buck, C. E., Burr, G. S., Edwards, R. L., Friedrich, M., Grootes, P. M., Guilderson, T. P., Hajdas, I., Heaton, T. J., Hogg, A. G., Hughen, K. A., Kaiser, K. F., Kromer, B., McCormac, F. G., Manning, S. W., Reimer, R. W., Richards, D. A., Southon, J. R., Talamo, S., Turney, C. S. M., van der Plicht, J. & Weyhenmeyer, C. E. (2009). IntCal09 and Marine09 radiocarbon age calibration curves, 0-50,000 years cal BP. *Radiocarbon* **51**, 4, 1111-1150.

Reuss, A. E. (1850). Neue Foraminiferen aus den Schichten des österreichischen Tertiärbeckens. Denkschriften der Kaiserlichen Akademie der Wissenschaften. 1, 365-390.

Reuss, A. E. (1851). Ueber die fossilen Foraminiferen und Entomostraceen der Septarienthone der Umgegend von Berlin. *Zeitschrift der Deutschen Geologischen Gesellschaft.* 3, 49-92.

Schwager, C. (1866). Fossile Foraminiferen von Kar Nikobar. Reise der Österreichischen Fregatte Novara um die Erde in den Jahren 1857, 1858, 1859 unter den Befehlen des Commodore B. von Wüllerstorf-Urbair. Geologischer Theil (Zweite Abtheilung, Paläontologische Mittheilungen), 2, 187-268.

Seguenza, G. (1862). Prime ricerche intorno ai rizopodi fossili delle argille pleistoceniche dei dintorni di Catania. *Atti della Accademia Gioenia di Scienze Naturali in Catania.* ser. 2, 18, 85-125.

Silvestri, A. (1896). Foraminiferi pliocenici della provincia di Siena. Parte I. Memorie dell'Accademia Pontificia dei Nuovi Lincei 12, 224 p.

Stewart, R. E., Stewart, K. C. (1930). Post-Miocene foraminifera from the Ventura Quadrangle, Ventura County, California; twelve new species and varieties from the Pliocene. Journal of Paleontology, 4, 60-72.

Stuiver, M., Reimer, P. J. & Reimer, R. W. (2005). CALIB 5.0. (WWW program and documentation: http://calib.qub.ac.uk/calib/, access 01/23/2013).

Thalmann, H. E. (1939). Bibliography and index to new genera, species and varieties of foraminifera for the year 1936, Journal of Paleontology 13, 425-465.

Voloshinova, N. A. (1958). On new systematics of the Nonionidae (Russian) Microfauna of the U.S.S.R., Col. 9, 115: 117–223.

Walker, C., Jacob, E., in: Kanmacher, F. (1798). Essays on the microscope. The Second Edition, with considerable additions and improvements. Dillon & Keating, London. 724 p..

Williamson, W. C. (1848). On the Recent British species of the genus *Lagena*. Annals and Magazine of Natural History, 1, 1-20.

Williamson, W.C. (1858). On the recent Foraminifera of Great Britain. The Ray Society, London. 107 p.
